# Ecological drivers of genetic connectivity for African malaria vectors *Anopheles gambiae* and *An. arabiensis*

**DOI:** 10.1038/s41598-020-76248-2

**Published:** 2020-11-17

**Authors:** Elizabeth Hemming-Schroeder, Daibin Zhong, Maxwell Machani, Hoan Nguyen, Sarah Thong, Samuel Kahindi, Charles Mbogo, Harrysone Atieli, Andrew Githeko, Tovi Lehmann, James W. Kazura, Guiyun Yan

**Affiliations:** 1 Department of Ecology and Evolutionary Biology and Program in Public Health, University of California, Irvine, CA 92617 USA; 2 Center for Global Health Research, Kenya Medical Research Institute, Kisumu, Kenya; 3 School of Pure and Applied Sciences, Pwani University, Kilifi, Kenya; 4 School of Public Health and Community Development, Maseno University, Kisumu, Kenya; 5 Laboratory of Malaria and Vector Research, National Institutes of Health, Bethesda, MD 20892 USA; 6 Present Address: Center for Global Health and Diseases, Case Western Reserve University, Cleveland, OH 44106 USA

**Keywords:** Ecological genetics, Malaria

## Abstract

*Anopheles gambiae* and *An. arabiensis* are major malaria vectors in sub-Saharan Africa. Knowledge of how geographical factors drive the dispersal and gene flow of malaria vectors can help in combatting insecticide resistance spread and planning new vector control interventions. Here, we used a landscape genetics approach to investigate population relatedness and genetic connectivity of *An. gambiae* and *An. arabiensis* across Kenya and determined the changes in mosquito population genetic diversity after 20 years of intensive malaria control efforts. We found a significant reduction in genetic diversity in *An. gambiae*, but not in *An. arabiensis* as compared to prior to the 20-year period in western Kenya. Significant population structure among populations was found for both species. The most important ecological driver for dispersal and gene flow of *An. gambiae* and *An. arabiensis* was tree cover and cropland, respectively. These findings highlight that human induced environmental modifications may enhance genetic connectivity of malaria vectors.

## Introduction

*Anopheles gambiae s.l.* are the primary vectors of human malaria in sub-Saharan Africa, a disease responsible for 405,000 deaths worldwide annually, with around 90% occurring in Africa^1^. While the species commonly occupy similar ecological niches, *An. gambiae s.s.* (hereafter referred to as *An. gambiae)* are generally associated with more humid environments, whereas *An. arabiensis* have a higher tolerance for drier environments^2,3^. Another notable difference between the two species is that *An. gambiae* are highly anthropophagic^4,5^, whereas *An. arabiensis* are more catholic in their feeding behavior^6^. Since mosquitoes primarily disperse to seek blood meals and oviposit^7^, these differences in habitat and feeding preferences may result in ecological variables differentially driving the dispersal patterns of these two malaria vectors. These differences potentially lead to a complex system influencing malaria parasite spread. Knowledge of how geographical factors influence the dispersal of malaria vectors can help in efforts to contain insecticide resistance, planning effective vector control interventions, and identifying potential areas susceptible to parasite re-introduction from infected mosquitoes following antimalarial interventions^8^.

Organism dispersal patterns and population connectedness can be inferred from measuring genetic relatedness among populations or gene flow. While studies have reported genetic differentiation between *An. arabiensis* populations, neither physical barriers nor geographic distance have been identified as factors responsible for *An. arabiensis* population structuring^9–12^. Likewise, geographical distance alone does not appear to be a barrier to gene flow among populations of *An. gambiae*, as Lehmann et al.^13^ found high gene flow between populations in Kenya (East Africa) and Senegal (West Africa). However, *An. gambiae* populations were found to be highly differentiated between western Kenya and eastern Kenya^12^. The eastern arm of the Great Rift Valley, which bisects Kenya, has been speculated to be the cause of genetic differentiation in *An. gambiae* populations due to its surrounding low temperatures and arid conditions making it inhospitable to agriculture, and as such, lacks human settlements^12,14^. Since the eastern arm of the Great Rift Valley is characterized by several attributes, such as low temperatures, low precipitation, as well as low human population density^12^, these factors cannot be disentangled for driving population structure of *An. gambiae* using traditional population genetics*.*

However, using a landscape genetics approach, we can test the impacts of ecological variables on organism dispersal to parse out the importance of key variables influencing malaria vector dispersal^8,15^. Broadly, the approach involves inferring population movement from the distribution of genetic markers, quantifying the distribution of ecological factors hypothesized to drive dispersal, and statistically testing the relationships between genetic variation and landscape heterogeneity^15–17^. These inferences enable us to identify potential hotspot areas of disease movement for targeted public health interventions and containment of disease and drug and insecticide resistance^8^. For example, this approach has been used to identify dispersal corridors for *Ae. albopictus* mosquitoes in the United States, which were found to be primarily highways and non-forested areas^18^. Moreover, landscape genetics has been used to study infectious diseases, including chronic wasting disease^19^, rabies^20,21^, hantavirus^22^, H5N1 avian influenza^23^, and malaria^24,25^. The objective of this study is to use a landscape genetics approach to test the hypothesis that genetic connectivity of *An. gambiae* and *An. arabiensis* is influenced by environmental, landscape, or social factors, as opposed to geographic distance alone. By testing relationships between population genetic structure of malaria vectors and ecological factors, we can parse out confounding factors and determine the importance of key variables influencing malaria vector dispersal^8,15^.

## Results

*Anopheles gambiae s.l.* larvae were collected in 2014–2015 from thirteen sites across Kenya, which fall within four distinct geographical areas: western Kenya lowlands, western Kenya highlands, Great Rift Valley, and coastal Indian Ocean (Supplementary Table 1; Supplementary Fig. 1). *An. arabiensis* specimens included in analyses originated from ten populations in western Kenya, Great Rift Valley and coastal Indian Ocean (total individuals = 357); whereas, *An. gambiae* specimens included in analyses originated from six populations in western Kenya only (total individuals = 254). To assess genetic diversity and structure of vector populations, six microsatellite loci were genotyped in *An. arabiensis* and five microsatellites were genotyped in *An. gambiae* specimens (Supplementary Table 2).

### Genetic diversity analysis

**Figure 1:**
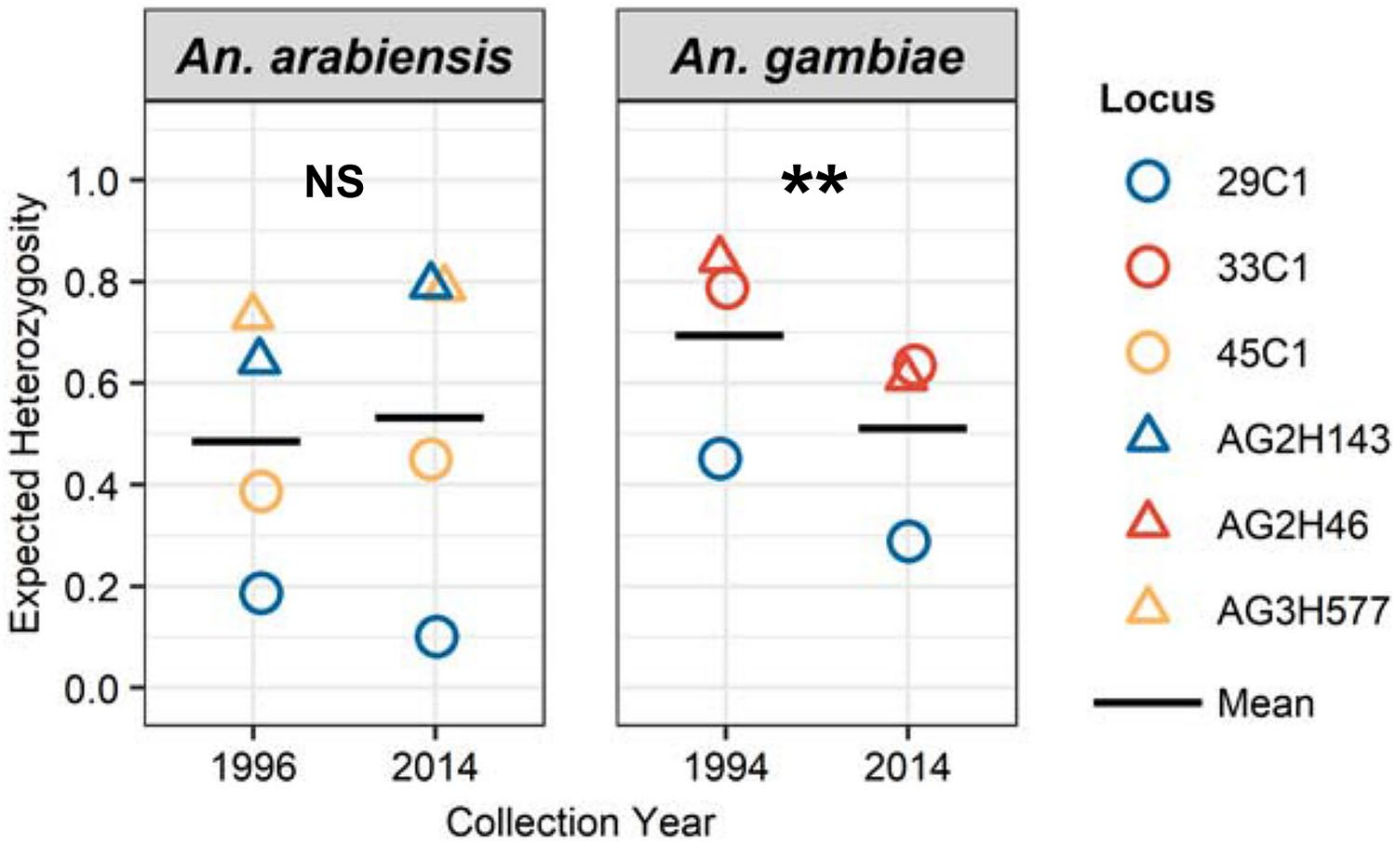
Comparison of genetic diversity (expected heterozygosity) between 1994*/*1996 and 2014 from nearby study sites within the Lake Victoria basin area of western Kenya for *Anopheles arabiensis* and *An. gambiae*. **P < 0.01; NS indicates *P* > 0.05 by paired t-tests. Data from 1996 was obtained from Kamau et al.^12^. Data from 1994 was obtained from Lehmann et al.^26^.

For *An. arabiensis*, a measure of genetic diversity, expected heterozygosity (H_E_), ranged from 0.461 in an Indian Ocean coastal site to 0.723 in an Indian Ocean coastal study site (Supplementary Table 3). H_E_ did not vary significantly among regions (Supplementary Fig. 2). Mean allelic richness (A_R_) ranged from 3.46 in an Indian Ocean coastal site to 6.24 in a western lowland site (Supplementary Table 3). For *An. gambiae,* H_E_ and A_R_ were generally higher in the highland sites (H_E_:0.588–0.653; A_R_:3.74–5.87) compared to the western Kenya lowland sites (H_E_:0.503–0.584; A_R_:3.57–4.90) (Supplementary Fig. 2; Supplementary Table 3). An overall heterozygote deficiency was detected across all populations (*P* < 0.05) for both species. There was no evidence indicating linkage disequilibrium at any locus across all population for *An. arabiensis* (*P* > 0.05). By population, there was significant linkage disequilibrium in *An. arabiensis* detected at two study sites in western Kenya (HB: 45C1/29C1 and AG2H143/AG3H577; KB: 29C1/AG3H577), one study site in the Great Rift Valley (MT: AG3H249/AG2H143), and two study sites in Indian Ocean coastal Kenya (JU: AG2H46/AG2H143; KK: AG2H46/29C1). For *An. gambiae,* linkage disequilibrium was detected in two locus pairs across all populations (AG2H143/29C1 and AG2H143/AG3H577; *P* < 0.05). By population, there was significant linkage disequilibrium in *An. gambiae* at three study sites in the western lowlands (PB: AG2H143/AG3H577, AG2H46/AG2H143, and AG2H46/AG3H577; KN: AG2H143/29C1; HB: AG2H143/29C1 and AG2H143/AG3H577) and one study site in the western highlands (EE: AG2H46/29C1). Since all of the locus pairs observed to be in linkage disequilibrium for both species do not occur in the same chromosomal region (Supplementary Table 2), this result suggests the possibility that random drift or non-random mating are leading to the observed linkage disequilibrium in *An. arabiensis* and *An. gambiae.* Compared to previously published H_E_ values for *An. arabiensis* samples collected from nearby study sites within the Lake Victoria basin area of Western Kenya in 1996^12^, we observed no significant change in overall H_E_ between 1996 and 2014 (Fig. 1; *P* = 0.417; paired t-test). In comparison to previously published H_E_ values for *An. gambiae* samples collected in 1994^26^, overall H_E_ was significantly lower in 2014 (Fig. 1; *P* = 0.002, paired t-test).

### Population structure and migration rates

**Figure 2:**
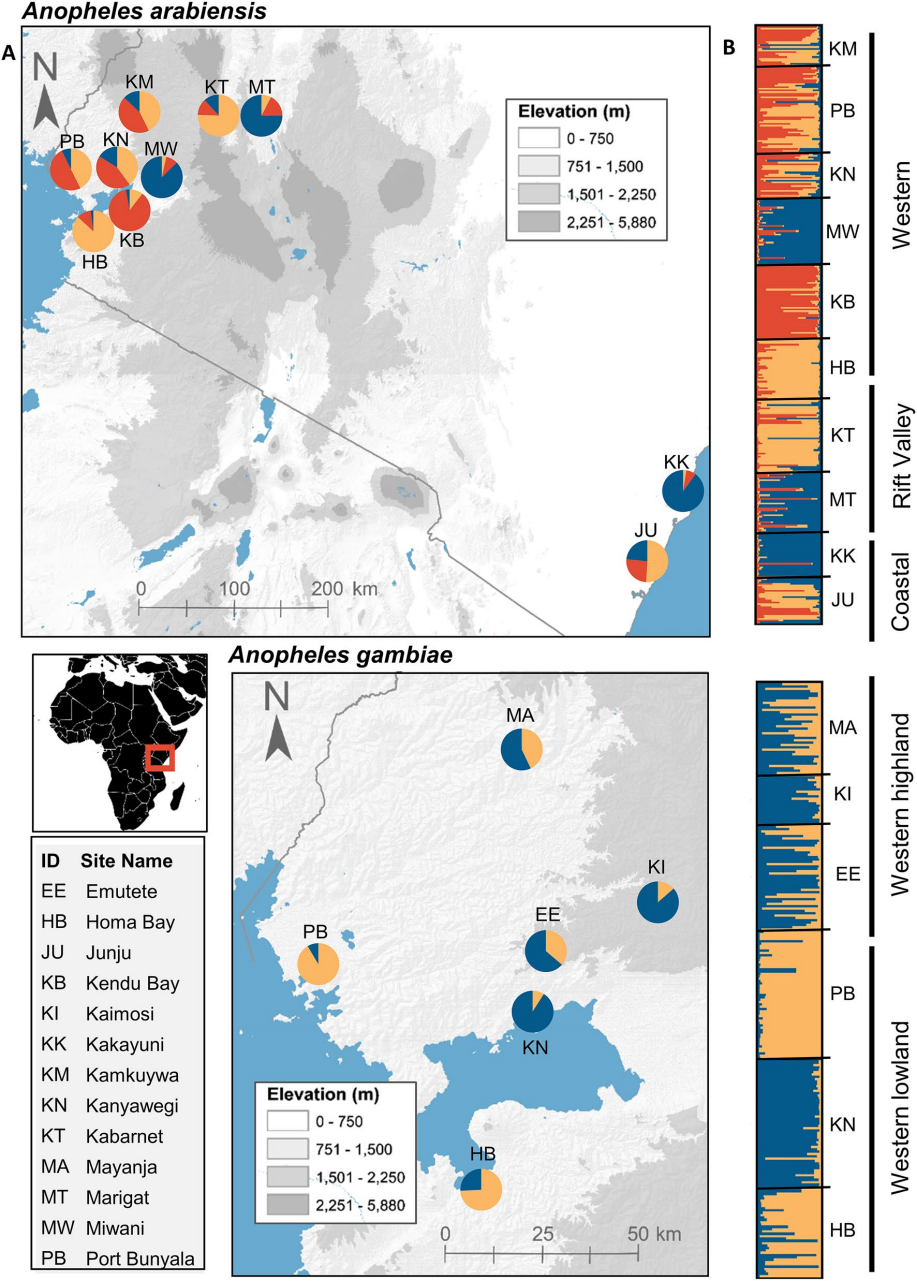
Inferred population structure estimated by STRUCTURE among *Anopheles arabiensis* (K = 3) and *An. gambiae* (K = 2). (**A**) Map of mean ancestral coefficients by study location. Pie chart color indicates proportion of population assigned to each ancestral cluster. (**B**) Individual level ancestral coefficients. Individuals are represented as rows. Horizontal black lines indicate division in sampling location and vertical black bars indicates region of sampling location. The most probable cluster is indicated by color. Within each individual, the extent of the component colors indicates the magnitude of the membership coefficient corresponding to each cluster. The background elevation maps were created from Shuttle Radar Topography Mission (SRTM) data in ArcMap 10.6.1 (https://desktop.arcgis.com/en/arcmap/).

*For An. arabiensis*, we identified three clusters consistently across ten runs in Structure (Fig. 2). Predominant cluster membership varied between coastal Kenya and the other two regions. The AMOVA indicated that 12.77% of variation occurred among groups, which were populations grouped by their predominant cluster (F_CT_ = 0.128; *P* < 0.001). Variation within populations (79.7%; F_ST_ = 0.203; *P* < 0.001) and variation occurred among populations within groups (7.49% F_SC_ = 0.086; *P* < 0.001) were also significant. Fo*r An. gambiae,* we identified two clusters consistently across ten runs in Structure (Fig. 2). Predominant cluster membership varied between the western lowland and western highland regions. The AMOVA indicated that 6.22% of variation occurred among groups, which were populations grouped by their predominant cluster (F_CT_ = 0.062; *P* = 0.058). Variation within populations (86.9%; F_ST_ = 0.131; *P* < 0.001) and variation occurred among populations within groups (6.84% F_SC_ = 0.073; *P* < 0.001) were significant. Based on a linear mixed effects model with maximum likelihood population effects (MLPE), populations did not conform to an isolation-by-distance model for either species (*P* > 0.05; Supplementary Fig. 3).

**Figure 3:**
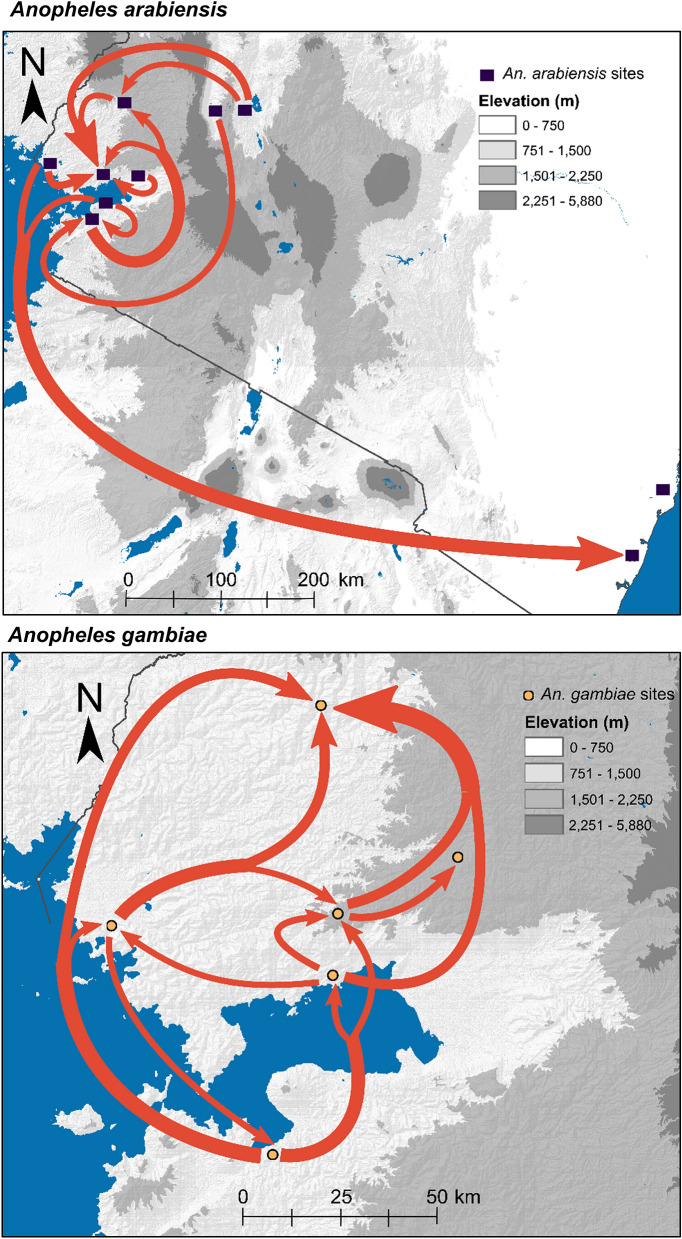
Migration directionality and intensity among *Anopheles arabiensis* and *An. gambiae* populations in Kenya*.* The intensity of gene flow is indicated by the width of migration arrows. Only migration rates with median M > 18 are indicated on the map. The background elevation maps were created from Shuttle Radar Topography Mission (SRTM) data in ArcMap 10.6.1 (https://desktop.arcgis.com/en/arcmap/).

Historical migration rates between vector populations were evaluated by analysis in Migrate-N, and the highest directional migration rates (migration rate [M] > 18) were visualized (Fig. 3).Analysis of past migration rates for *An. arabiensis* revealed that frequent migrations (M > 18) occurred between populations across regions, as well as between populations within regions (Fig. 3). The Great Rift Valley was a source of migration for four sites in western Kenya. Frequent migrations were common among sites within western Kenya. For *An. gambiae,* lowland populations were more often sources for migration than highland populations. Frequent migrations occurred most often from a lowland population to a highland population. Migration rates for all pairwise population comparisons are available in Supplementary Table 4.

### Landscape genetic analysis

We used landscape genetics to test for associations between landscape factors and gene flow between populations. We created landscape resistance raster files for the following variables, which we hypothesized to potentially influence gene flow of the studied malaria vector species: annual mean temperature, annual precipitation, percent tree cover data, presence or absence of croplands or cropland/natural vegetation mosaics, human population density, and road proximity (Table 1). Landscape genetic analysis was conducted using two measures of genetic distance, F_ST_ and D_PS_. Since the results from both analyses did not vary substantially, F_ST_ analyses are presented here, and D_PS_ analyses are presented in the supplementary material (Supplementary Table 5; Supplementary Fig. 4). Notably, the genetic distance metrics were highly correlated (*P* < 0.0001 for both species). For *An. arabiensis,* cropland was the top single-surface model in explaining population genetic structure of *An. arabiensis* (Table 2). The cropland model had the lowest ΔAIC (0.000), third lowest average rank (2.335), and highest percentage for being the top model across 1000 bootstrap iterations (34.8%). For *An. gambiae,* the tree cover model was the top model in explaining population genetic structure, which had the lowest ΔAIC (0.000), second lowest average rank (1.891), and highest percentage for being the top model across bootstrap iterations (40.3%).

**Table 1:** Predictor variables used for landscape genetic analysis.

	Variable	Source
Environmental	Average temperature	WorldClim BIO1
	Annual precipitation	WorldClim BIO12
Landscape	Percent tree cover	NASA MOD44B
	Cropland	NASA MOD12Q
Social	Human population density	Worldpop
	Road proximity	OpenStreetMap

**Table 2:** Model selection results of for linear mixed-effects models optimized on pairwise genetic differentiation (F_ST_) in ResistanceGA.

Model	K	Avg. ΔAIC	Avg. rank	Top model (%)
***Anopheles arabiensis***				
(1) Cropland	2	0	2.335	34.8
(2) Temperature	2	1.865	1.878	30.0
(3) Precipitation	2	2.257	2.220	26.3
(4) Geographic distance	1	3.526	3.567	8.9
***Anopheles gambiae***				
(1) Tree cover	2	0	1.891	40.3
(2) Temperature	2	1.520	1.778	35.2
(3) Precipitation	2	3.428	2.635	19.3
(4) Geographic distance	1	6.271	3.696	5.2

K, number of parameters in the mixed effects model; Avg., averaged over 1000 bootstrap iterations; ΔAICc, Difference in Akaike information criterion from the lowest AIC model; rank, model ranking; top model, percentage of the bootstrap iterations that a model was the top model.

**Figure 4:**
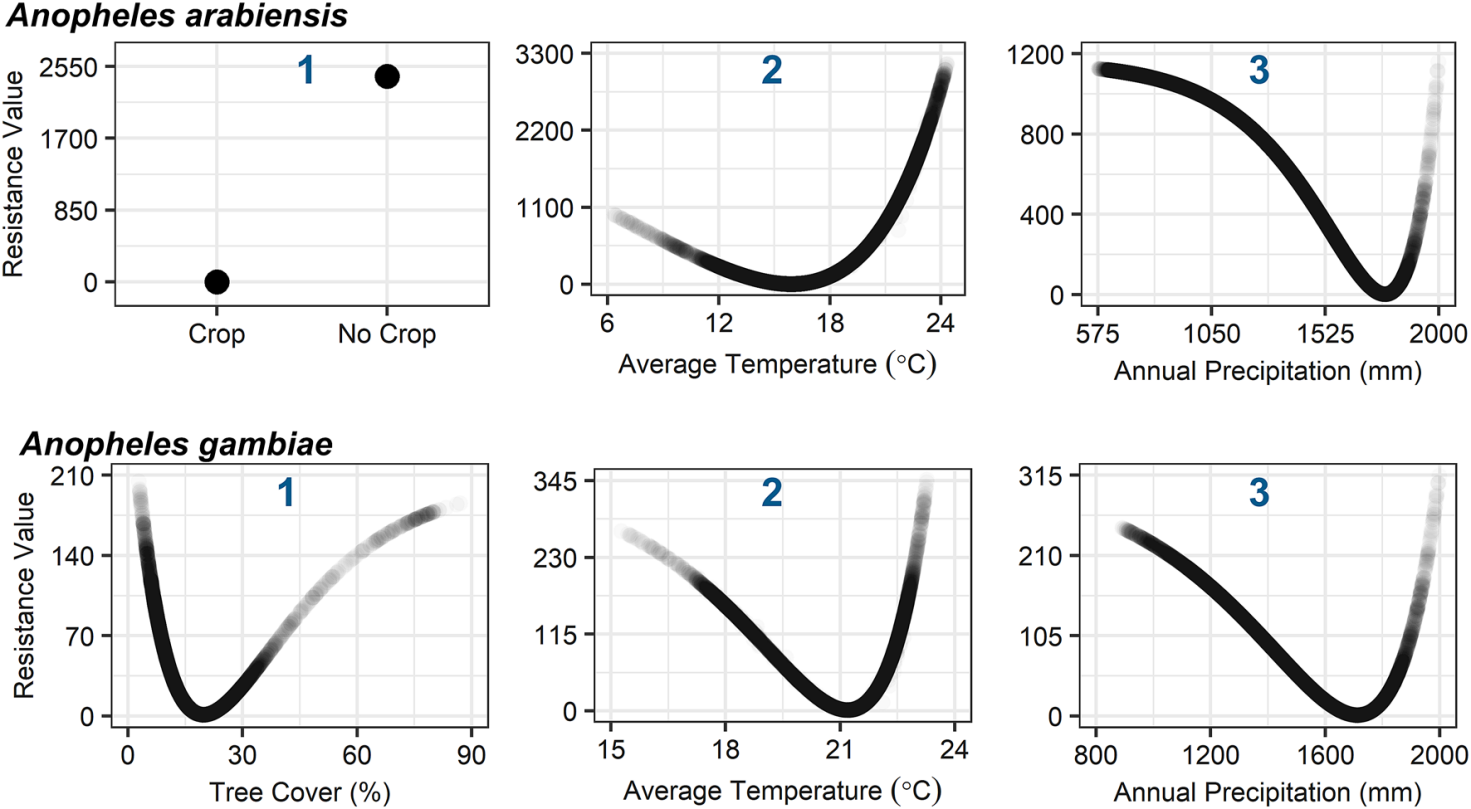
Response curves signifying the relationship between ecological variables and landscape resistance to gene flow in the three highest performing single-surface models for *Anopheles gambiae* and *An. arabiensis*. Blue number in plot indicates model ranking by Avg. ΔAIC value.

**Figure 5:**
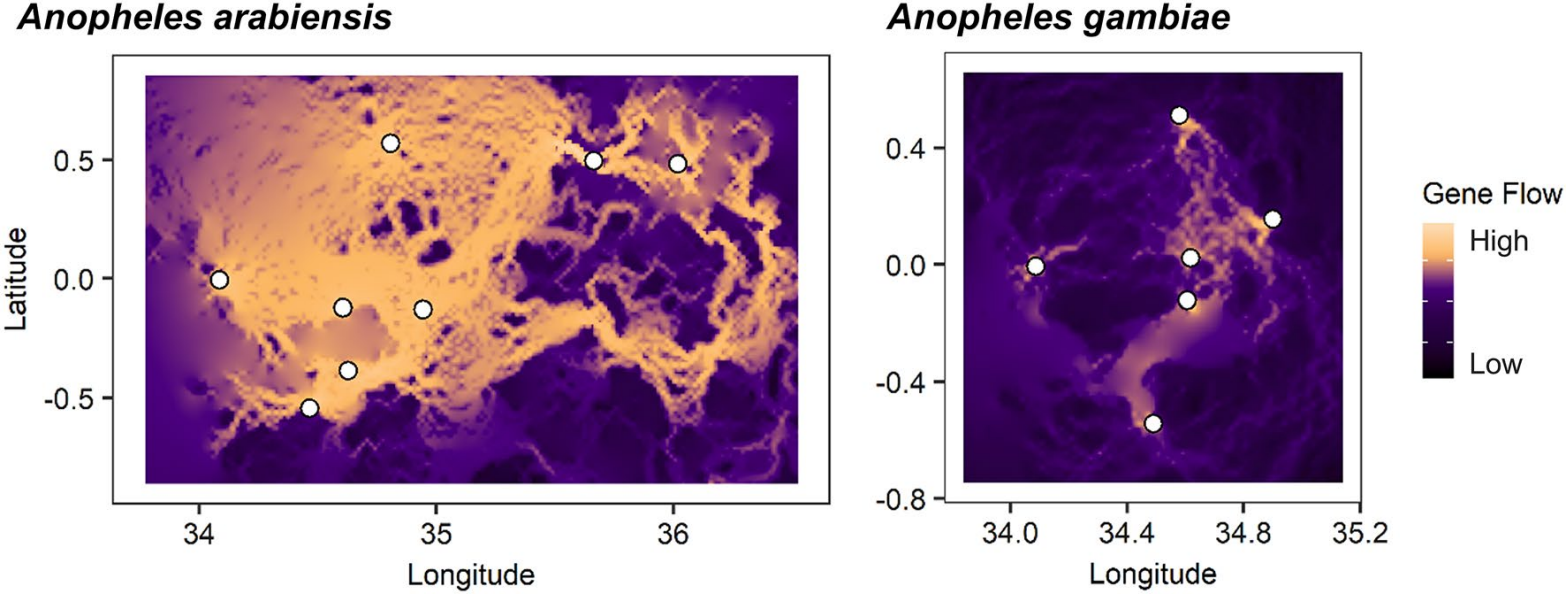
Gene flow map based on highest performing landscape resistance model for *Anopheles gambiae* and *An. Arabiensis. *Black circles indicate sampling locations. Yellow color indicates areas of high hypothesized gene flow between sampling locations. For *An. arabiensis,* map is based on the cropland model. For *An. gambiae,* map is based on the tree cover model. The maps were created in R 3.6.0 (https://www.r-project.org/rdata) with output currents from Circuitscape 4.0 (https://circuitscape.org/) based on the highest performing landscape resistance models.

To determine the relationship between model factors and landscape resistance to gene flow, response curves were generated (Fig. 4). In the highest performing model for *An. arabiensis*, cropland, areas with croplands or cropland/natural vegetation mosaics had a low resistance to gene flow. Average temperature was the next highest performing model. An average temperature of 15.9 °C had the lowest landscape resistance to gene flow. Areas with an average temperature exceeding 23.3 °C had the highest landscape resistance to gene flow (highest 30% resistance values). For the third highest performing model, landscape resistance to gene flow was lowest in areas with annual precipitation 1530.8–1914.8 mm (lowest 30% resistance values) and highest in areas with annual precipitation less than 1230.8 mm and greater than 1970.8 mm. In the highest performing model for *An. gambiae,* the lowest landscape resistance values occurred in areas with 9.2–38.2% tree cover and the highest landscape resistance values occurred in areas with less than 4.9% tree cover and greater than 61.2% tree cover. For the second highest performing model, the lowest landscape resistance occurred in areas with an average annual temperature between 18.9 and 22.6 °C and the highest landscape resistance values occurred in areas with an average temperature less than 16.1 °C and greater than 23.0 °C. For the third highest performing model, the lowest landscape resistance to gene flow occurred in areas with 1401.5–1890.8 mm annual precipitation and the highest landscape resistance to gene flow occurred in areas with less than 1017.5 mm and greater than 1963.0 mm annual precipitation. Our visualization of gene flow between study sites based on the highest performing models, cropland for *An. arabiensis* and tree cover for *An. gambiae,* identifies likely pathways for gene flow between study sites (Fig. 5).

## Discussion

Using a landscape genetics framework to test hypotheses related to whether environmental, landscape, or social factors predominantly influence the population structures of *An. gambiae* and *An. arabiensis,* we found that landscape and environmental factors primarily influence the population structures of these species in western Kenya. For *An. gambiae*, tree cover was the most important factor shaping population structure. While, for *An. arabiensis,* presence of cropland was the highest performing explanatory factor. These ecological drivers of gene flow may help to explain broad-scale population structuring patterns previously observed.

In this study, we observed a significant reduction in diversity (H_E_) in *An. gambiae* from 1994^26^ to 2014 (this study), which were collected from neighboring populations within the Lake Victoria basin area of western Kenya. For *An. arabiensis,* we did not observe a comparable reduction in H_E_ in between similar time points, 1996^12^ to 2014 (this study). Notably, we do not report on diversity from within the 18 to 20-year period, so we cannot describe how diversity may have fluctuated within this time frame. With that said, these findings of reduced H_E_ in *An. gambiae* are consistent with a population decline*,* which has been observed throughout much of East Africa in response to mass distribution and increased coverage of insecticide-treated bed nets (ITNs)^27–32^. Conversely, the maintenance of H_E_ in *An. arabiensis* suggests that *An. arabiensis* populations have remained relatively more stable despite the high coverage of ITNs in Kenya. *Anopheles gambiae* are known to be more susceptible to malaria interventions than *An. arabiensis* due to their preference for feeding on humans and resting indoors^4,5^. Whereas *An. arabiensis* are more catholic in their feeding behavior, taking blood meals from both human and non-human hosts, especially cattle^33–35^, as well as more commonly feed and rest outdoors^27,28,36,37^. These findings highlight the role that *An. arabiensis* may play in maintaining residual malaria transmission in sub-Saharan Africa^28,38^ and the need for alternative intervention strategies to target this species. Moreover, understanding how ecological features influence *An. arabiensis* dispersal will likely become increasingly important to interrupting residual malaria transmission.

Neither *An. arabiensis* nor *An. gambiae* conformed to an isolation-by-distance model in this study*.* These findings are consistent with previous studies^9–13^, which suggests the possibility that factors aside from geographic distance alone drive gene flow, such as climate, landscape, or social features. Additionally, for *An. arabiensis,* we found frequent past migration across regions of Kenya. This finding is also consistent with previous studies and may be in part due to a past range expansion, facilitated by the expansion of human settlement and agriculture, rather than large-scale, contemporary cross-country migrations^39–41^. For *An. gambiae,* most frequent migrations occurred from lowland to highland populations. Nonetheless, we did find significant population structuring, indicating the presence of gene flow barriers for *An. arabiensis* and *An. gambiae* in Kenya*.* Notably, F_ST_ values for both species were on average higher than those observed in the 1990s^12,26^, which suggests that population connectivity has reduced since then. This pattern of reduced connectivity may be potentially confounded by the recent history of vector control. With that said, these results together suggest the possibility that factors aside from distance alone drive gene flow patterns of malaria vectors in Kenya.

We tested the hypothesis that low human population densities and scant agriculture between *An. gambiae* populations provide a barrier to gene flow^12,14^. This notion was postulated since populations of *An. gambiae* between western Kenya and eastern Kenya were found to be more distinct than between western Kenya and Senegal in western Africa, despite that the two countries are separated by more than 5000 km^12^, whereas western Kenya and coastal Kenya populations are only 700 km apart^12^. The lack of human settlement on the high plateaus of the eastern arm of the Great Rift Valley, which bisects Kenya, was thought to explain the population structuring pattern, since unlike the eastern arm of the rift, human agricultural activity occurs in a broad band across the area between Senegal and western Kenya^12^. However, we provide evidence that tree cover primarily explains gene flow patterns in western Kenya rather than human population densities or agriculture. Both low (< 5%) and high (> 61%) tree cover were found to be gene flow barriers for *An. gambiae* in western Kenya. Whereas an intermediate tree cover of 9–38% was associated with enhanced gene flow for *An. gambiae.* This finding is consistent with a previous study in Kenya, which found that a canopy cover of 26% was associated with the presence of the *An. gambiae* complex*,* while a canopy cover of 44% was associated with the absence of the *An. gambiae* complex^42^*.* Moreover, this finding may also be consistent with the broad-scale population structuring observed across Africa^12,14^, as unlike with the western arm of the Great Rift Valley, a wide swath of land to the East of the eastern arm of the Great Rift Valley is characterized by short vegetation and a tree cover < 5% (Supplementary Fig. 5).

While populations of *An. gambiae* across the eastern arm of the rift were previously found to be highly genetically differentiated, lower differentiation was found between populations of *An. arabiensis* in this and other studies^12^. This result suggested that different factors drive gene flow patterns for *An. arabiensis* than for *An. gambiae* despite that the species commonly occupy similar ecological niches^2,3^. We provide evidence in support of this conjecture, as unlike for *An. gambiae,* a low percent tree cover was not a barrier to gene flow for *An. arabiensis,* but rather, the absence of cropland was the primary barrier to gene flow. Notably, however, the presence and absence of cropland does not explain the lack of population structuring between western and eastern Kenya observed by Kamau et al. in the late 1990s^12^_,_ as western and eastern Kenya are not connected by cropland areas (Supplementary Fig. 5). That this cropland model does not explain population structuring patterns outside of western Kenya may be explained by previous observations that *An. arabiensis* is more of a climate generalist than *An. gambiae*^38^. *Anopheles arabiensis* are found across a very diverse environmental range, which ultimately allows the survival of the species in many locations in Africa^38^. Moreover, *An. arabiensis* are known to be relatively resilient to drastic environmental changes through both natural causes and control measures^43^. Lastly, since *An. arabiensis* are able to withstand more arid conditions than *An. gambiae*^44^, it is possible that other environmental indicators which were not evaluated in this study explain gene flow patterns, such as maximum temperature. Since *An. arabiensis* may not be as constrained by environmental heterogeneity, as well as are less susceptible to traditional malaria interventions, controlling residual malaria transmission may prove more challenging and require additional, alternative interventions.

The importance of tree cover in shaping population structure of *An. gambiae* and cropland for *An. arabiensis* suggests the significant role that land cover and land use plays in providing suitable microhabitats for larvae to develop. This notion is underscored by the fact that the tree cover model outperformed models based on environmental and social factors in *An. gambiae,* and that cropland was the top model for *An. arabiensis*. Too much tree cover may lead to a lack of sunlight and lower temperatures, which slows or inhibits the growth of larvae^42^. Whereas a lack of tree cover may lead to or be indicative of conditions that are too dry, potentially leaving adult *An. gambiae* susceptible to desiccation^44,45^. Whereas *An. arabiensis* are more desiccation tolerant^44^, and as such, can thrive in drier environments. These findings of high tree cover hindering gene flow of *An. gambiae* highlight the effect that deforestation may have in promoting gene flow of malaria vectors. Further, deforestation for agricultural purposes may promote gene flow of *An. arabiensis*, as cropland areas may enhance gene flow. Thus, deforestation may increase invasion risk of novel malaria vector and parasite genotypes to surrounding areas, potentially also enhancing the spread of insecticide resistant vectors and drug resistant parasites.

This study has several limitations. First, the landscape genetic analysis included genetic data from a single time point. It is not clear whether these spatial trends would be consistent across time. Second, we only examined relationships between genetic distance and ecological variables at one spatial scale. Patterns driving gene flow for these species may vary across spatial scales, i.e. one factor may be more important at a finer scale than at a coarser scale^8^. Third, the genetic data used for this study was based on five to six microsatellite loci per species, which may provide a limited sample of the genome. Future studies capturing a larger proportion of the genome may improve the resolution of population structure assignment. Finally, this landscape genetics analysis only examined populations in western Kenya. It is likely that different factors drive gene flow patterns depending on the ecological setting, and thus the patterns identified here may differ from those in another region. Thus, the generalizability of these findings to different scales and regions is unknown and requires further investigation. With that said, this study has sought to extend our understanding of local malaria transmission settings in Kenya. Further, this approach may be broadly applicable for investigating factors driving gene flow of malaria vectors in other geographic regions, as well as for additional studies in Kenya.

To conclude, we found that corridors for *An. gambiae* in western Kenya are most likely to be areas of moderate tree cover (9–38%). Potential corridors for *An. arabiensis* are areas with cropland or cropland/natural vegetation mosaics. These findings underscore that human induced land cover and land use modifications may enhance connectivity of these species. Specifically, agricultural deforestation may promote dispersal and gene flow for both species. Understanding the factors that limit and promote movement (gene flow) of malaria vectors can help us to more effectively deploy interventions to maintain vector control by identifying areas susceptible to invasion from neighboring mosquito populations. Moreover, this knowledge can be leveraged to help limit the spread of malaria parasites by mosquitoes from nearby areas, as well as contain insecticide resistance^8^. Finally, knowledge of factors influencing malaria vector dispersal are likely to become increasingly important as malaria transmission becomes increasingly heterogeneous^46^.

## Methods

### Sample collection

*An. gambiae s.l.* larvae were collected between May 2014 and January 2015 from thirteen sites across Kenya (Supplementary Fig. 1). These sites fall within four distinct geographical areas: western Kenya lowlands, western Kenya highlands, Great Rift Valley, and coastal Indian Ocean (Supplementary Table 1. Larvae were collected using a standard mosquito dipper. No more than five larvae were collected per habitat (pool of water) to reduce potential bias from collecting mosquito full siblings^47^. Larvae in a given study site were collected from between 14 and 53 habitats within a 1.5 km diameter area. Collected larvae were stored in 100% ethanol until DNA purification. Genomic DNA was extracted using standard ethanol extraction procedures with phenol:chloroform^48^. DNA was eluted into 20 µl of TE buffer. Then, DNA was quantified using a NanoDrop 8000 Spectrophotomer and diluted to a concentration of 1 µg/1 µl sterile water. We identified *An. arabiensis* and *An. gambiae* species within the *An. gambiae* s.l. complex using a ribosomal DNA polymerase chain reaction (PCR) assay^49^.

### Microsatellite genotyping

Six microsatellite loci were selected for genotyping *An. gambiae* and five loci were selected for *An. arabiensis* based on evidence of polymorphism in previous studies, reliable amplification, and distribution across chromosomes (Supplementary Table 2)^50^. We used the M13 tailed primer method to fluorescently label our primers^51^. Amplification was conducted in a total volume of 10 µl with 5 µl of 2 × DreamTaq Green PCR Master Mix (Thermo Fisher, USA), 0.5 µl of 10 µM primer (forward primer with M13 tail), and 1 µl of DNA template. Thermocycling conditions for *An. gambiae* and *An. arabiensis* were as follows: initial denature of 94 °C for 3 min, followed by 35 amplification cycles of 94 °C for 30 s, annealing temperature (Supplementary Table 3) for 30 s, and 72 °C for 45 s, and then a final extension of 72 °C for 6 min. PCR products were analyzed on an automated 4300 DNA analyzer (Li-Cor, Lincoln, NE), and alleles were quantified with the use of Gene ImagIR 4.33 software (Li-Cor).

### Population genetic analysis

To test whether population genetic diversity has been altered over the past two decades in the face of intense malaria vector control campaigns, namely the increase in use of insecticide-treated bednets (ITNs)^27–32^, we assessed measures of heterozygosity in Arlequin^52^. We then compared expected heterozygosity, a measure of genetic diversity, from western Kenya lowland specimens collected in this study to previously published expected heterozygosity values in nearby lowland populations of *An. arabiensis* and *An. gambiae,* which were collected in 1996^12^ and 1994^26^, respectively, prior to the scale-up in use of ITNs. Expected heterozygosity was averaged across nearby populations within the Lake Victoria basin area of western Kenya (n = 2 for *An. arabiensis-*1996 and *An. gambiae*-1994; n = 5 for *An. arabiensis-*2014; n = 3 for *An. gambiae-*2014) for each loci and then compared. We used paired t-tests to assess statistical significance between expected heterozygosity in pre-ITN scale-up populations (1994/1996) and post-ITN scale-up (2014) populations at matching loci. In addition, for populations from this study (2014), we tested for deviation from Hardy–Weinberg equilibrium and linkage disequilibrium (LD), independence of microsatellite loci, as well as estimated the inbreeding coefficient in Genepop 4.2^53,54^. We also estimated allelic richness at each study site in the R package *diveRsity*^55^.

### Population structure and migration rates

We estimated population structure using STRUCTURE v. 2.3.4, which uses a Bayesian algorithm to group samples into genetically distinct clusters^56^. We tested K = 1–7 for *An. gambiae* and K = 1–10 for *An. arabiensis*, with ten replicates for each K-level, an initial burn-in of 100,000, and then 500,000 Monte Carlo Markov Chain iterations. The program was run using an admixture model. ∆K was used to detect the number of K (clusters). The output data for the best estimate of K were analyzed using CLUMMP to calculate the mean cluster membership coefficients across multiple runs^57^. An analysis of molecular variance (AMOVA) was conducted in Arlequin to test for significance of genetic differentiation^52^. To test for isolation-by-distance, we used linear mixed effects models with the maximum likelihood population effects^58^ to fit pairwise geographic distance to F_ST_ measured in Genepop 4.2^53,54^. Gene flow frequency among populations was estimated for each species in Migrate-N v3.7.2 with the Brownian motion model and Bayesian inference search strategy^59^. Bidirectional gene flow between all pairwise population comparisons was considered. Four independent runs were conducted with a burn-in of 10^4^ steps, sampling increment of 100 steps, and 5,000 recorded steps in each chain for a total of 2 × 106 visited parameter values.

### Landscape genetic analysis

To test for hypothesized associations between landscape factors and gene flow between populations, we used a landscape genetic analysis approach. When testing the effects of environmental factors on gene flow between populations, between-site characteristics are of the greatest concern^60^. Hence, landscape resistance surfaces were created based on factors hypothesized to prevent or promote gene flow between sites. Since mosquitoes primarily disperse to seek blood meals and oviposit, as well as have physiological constraints regarding development and desiccation, we selected variables which influence these phenomena, i.e., the availability of blood meals and oviposition sites (larval habitats) and environmental suitability. Further, we selected variables that did not closely correlate with each other, which can lead to erroneous model selection results. All pairs of variables had a Pearson correlation coefficient <| 0.6 | (Supplementary Table 6). We created landscape resistance raster files in ArcGIS 10 using climate data (annual average temperature and annual precipitation) from WorldClim (BIO1 and BIO12)^61^, percent tree cover data from NASA (MOD44B)^62,63^, cropland from NASA (MOD12Q)^62,63^, human population density from WorldPop^64^, and road proximity from OpenStreetMap (Table 1). Specifically, tree cover and cropland variables were chosen because these factors may alter the local ambient temperature and humidity as well as the availability and nutrient content of larval habitats. The cropland raster file was created by reclassifying croplands and cropland/natural vegetation mosaics (IGBP land cover classification system) to “presence of cropland” and all other land cover types to “absence of cropland.” The cropland raster was categorized into two classes to limit the number of parameters estimated and to ensure classes were adequately represented in the landscape. The road proximity raster file was created using the proximity analysis tool in ArcGIS 10. All raster files were resampled to a grain size (resolution) of 2 km to balance computational efficiency. Landscape resistance distance among all pairs of sites was measured using the commuteDistance function in PopGenReport, which is based upon electrical circuit theory^65^. Resistance distance, defined as the effective resistance between a pair of nodes where all edges are replaced by analogous resistors, reflects the movement cost, as well as availability of alternative pathways, providing an advantage over the commonly used least cost path method^65^.

Genetic distance between populations was calculated as F_ST_ measured in Genepop 4.2^53,54^ and D_PS_ measured in PopGenReport^66^ (Supplementary Table 7; Supplementary Table 8). F_ST_ is a commonly used metric in population and landscape genetic studies, but relies on equilibrium assumptions, which may not be met. Whereas D_PS_ does not rely on any such theoretical assumptions. Evaluation of locus-specific effects to overall genetic distance (F_ST_) values indicated that F_ST_ values obtained from each individual locus positively correlates with overall F_ST_ for both species, but loci contribution to overall F_ST_ was not even, particularly with regard to the 29C1 locus for *An. arabiensis* (Supplementary Fig. 6). Since the 29C1 locus for *An. arabiensis* was an outlier in contributing to overall F_ST,_ pairwise F_ST_ was calculated both with and without the 29C1 locus. Notably, the omission of 29C1 in calculating overall F_ST_ did not substantially alter the isolation-by-distance analysis (Supplemental Fig. 6). Therefore, we used pairwise F_ST_ and D_PS_ values inclusive of 29C1 for landscape genetic analyses.

We used the R package ResistanceGA to unbiasedly optimize resistance surfaces to our genetic data^67^. This method has been demonstrated to have a low type 1 error rate for continuous and categorical surfaces, as well as a high correlation between true and optimized resistance surfaces in simulations^68^. Prior to optimizing our terrestrial resistance surfaces of interest (Table 1), we optimized a surface with lakes and land (no lakes) only to assign a resistance value to lakes. We then masked lakes in subsequent optimizations with the optimized resistance assignment value from the prior analysis, so that continuous land factors were optimized independent of lake features. Linear mixed effects models with the maximum likelihood population effects (MLPE) were used to fit optimized resistance surfaces to genetic data^58^. The three optimized resistance surfaces with the highest MLPE were then bootstrapped over 1000 iterations by subsampling 80% of the sample locations and refitting the MLPE model. Akaike information criterion (AIC) was used to compare model fitness to genetic data. We excluded eastern Kenya sites from landscape genetic analysis for *Anopheles arabiensis* due to the biases uneven sampling can introduce in analysis, as population sampling was not evenly distributed across the country^69^.

## Supplementary information

Supplementary Information.

## Data Availability

The authors declare that the data supporting the findings of this study are available within the paper and its supplementary information files (Supplementary File S1; Supplementary File S2).

## References

[1] World Health Organization (2019). World malaria report 2019.

[2] Wirtz RA, Burkot TR, Maudlin I, Sinha RC (1991). Detection of malarial parasites in mosquitoes. Advances in Disease Vector Research.

[3] Trape JF, Rogier C (1996). Combating malaria morbidity and mortality by reducing transmission. Parasitol. Today.

[4] Mala AO (2011). *Plasmodium falciparum* transmission and aridity: a Kenyan experience from the dry lands of Baringo and its implications for *Anopheles arabiensis* control. Malar. J..

[5] Macdonald G (1957). The Epidemiology and Control of Malaria.

[6] Gillies M, de Meillon B (1968). The Anophelini of Africa South of the Sahara (Ethiopian Zoogeographical Region).

[7] Service, M. W (1997). Mosquito (Diptera: Culicidae) dispersal—the long and short of it. J. Med. Entomol..

[8] Hemming-Schroeder E, Lo E, Salazar C, Puente S, Yan G (2018). Landscape genetics: a toolbox for studying vector-borne diseases. Front. Ecol. Evol..

[9] Ramsdale CD, Fontaine RE (1970). Ecological Investigations of *Anopheles gambiae* and *Anopheles funestus*.

[10] Charlwood JD, Vij R, Billingsley PF (2000). Dry season refugia of malaria-transmitting mosquitoes in a dry savannah zone of east Africa. Am. J. Trop. Med. Hyg..

[11] Aniedu I (1997). Dynamics of malaria transmission near two permanent breeding sites in Baringo district, Kenya. Indian J. Med. Res..

[12] Kamau L (1999). Analysis of genetic variability in *Anopheles arabiensis* and *Anopheles gambiae* using microsatellite loci. Insect Mol. Biol..

[13] Lehmann T (1996). Genetic differentiation of *Anopheles gambiae* populations from East and West Africa: comparison of microsatellite and allozyme loci. Heredity.

[14] Kamau L, Lehmann T, Hawley WA, Orago AS, Collins FH (1998). Microgeographic genetic differentiation of *Anopheles gambiae* mosquitoes from Asembo Bay, western Kenya: a comparison with Kilifi in coastal Kenya. Am. J. Trop. Med. Hyg..

[15] Storfer A (2007). Putting the ‘landscape’ in landscape genetics. Heredity.

[16] Biek R, Real LA (2010). The landscape genetics of infectious disease emergence and spread. Mol. Ecol..

[17] Storfer A, Murphy MA, Spear SF, Holderegger R, Waits LP (2010). Landscape genetics: Where are we now?. Mol. Ecol..

[18] Medley KA, Jenkins DG, Hoffman EA (2015). Human-aided and natural dispersal drive gene flow across the range of an invasive mosquito. Mol. Ecol..

[19] Blanchong JA (2008). Landscape genetics and the spatial distribution of chronic wasting disease. Biol. Lett..

[20] Cullingham CI, Kyle CJ, Pond BA, Rees EE, White BN (2009). Differential permeability of rivers to raccoon gene flow corresponds to rabies incidence in Ontario, Canada. Mol. Ecol..

[21] Côté H, Garant D, Robert K, Mainguy J, Pelletier F (2012). Genetic structure and rabies spread potential in raccoons: the role of landscape barriers and sex-biased dispersal. Evol. Appl..

[22] Guivier E (2011). Landscape genetics highlights the role of bank vole metapopulation dynamics in the epidemiology of Puumala hantavirus. Mol. Ecol..

[23] Carrel M, Wan XF, Nguyen T, Emch M (2011). Genetic variation of highly pathogenic H5N1 avian influenza viruses in Vietnam shows both species-specific and spatiotemporal associations. Avian Dis..

[24] Lo E (2017). Transmission dynamics of co-endemic *Plasmodium vivax* and *P. falciparum* in Ethiopia and prevalence of antimalarial resistant genotypes. PLoS Negl. Trop. Dis..

[25] Lo E (2017). Frequent spread of *Plasmodium vivax* malaria maintains high genetic diversity at the Myanmar–China Border, without distance and landscape barriers. J. Infect. Dis..

[26] Lehmann T (1997). Microgeographic structure of *Anopheles gambiae* in western Kenya based on mtDNA and microsatellite loci. Mol. Ecol..

[27] Bayoh MN (2010). *Anopheles gambiae*: historical population decline associated with regional distribution of insecticide-treated bed nets in western Nyanza Province, Kenya. Malar. J..

[28] Kitau J (2012). Species shifts in the *Anopheles gambiae* complex: do LLINs successfully control *Anopheles arabiensis*?. PLoS ONE.

[29] Mwangangi JM (2013). The role of *Anopheles arabiensis* and *Anopheles coustani* in indoor and outdoor malaria transmission in Taveta District, Kenya. Parasit. Vectors.

[30] Ototo EN (2015). Surveillance of malaria vector population density and biting behaviour in western Kenya. Malar. J..

[31] Sougoufara S, Harry M, Doucouré S, Sembène PM, Sokhna C (2016). Shift in species composition in the *Anopheles gambiae* complex after implementation of long-lasting insecticidal nets in Dielmo, Senegal. Med. Vet. Entomol..

[32] Hemming-Schroeder E (2018). Emerging pyrethroid resistance among *Anopheles arabiensis* in Kenya. Am. J. Trop. Med. Hyg..

[33] Githeko AK (1996). Some observations on the biting behavior of *Anopheles gambiae ss, Anopheles arabiensis,* and *Anopheles funestus* and their implications for malaria control. Exp. Parasitol..

[34] Massebo F, Balkew M, Gebre-Michael T, Lindtjørn B (2013). Blood meal origins and insecticide susceptibility of *Anopheles arabiensis* from Chano in South-West Ethiopia. Parasit. Vectors.

[35] Tirados I, Costantini C, Gibson G, Torr SJ (2006). Blood-feeding behaviour of the malarial mosquito *Anopheles arabiensis*: implications for vector control. Med. Vet. Entomol..

[36] Sinka ME (2010). The dominant *Anopheles* vectors of human malaria in Africa, Europe and the Middle East: occurrence data, distribution maps and bionomic précis. Parasit. Vectors.

[37] Charlwood JD (1995). The rise and fall of *Anopheles arabiensis* (Diptera: Culicidae) in a Tanzanian village. Bull. Entomol. Res..

[38] Drake JM, Beier JC (2014). Ecological niche and potential distribution of *Anopheles arabiensis* in Africa in 2050. Malar. J..

[39] Donnelly MJ, Cuamba N, Charlwood JD, Collins FH, Townson H (1999). Population structure in the malaria vector, *Anopheles arabiensis* Patton, in East Africa. Heredity.

[40] Donnelly MJ, Townson H (2000). Evidence for extensive genetic differentiation among populations of the malaria vector *Anopheles arabiensis* in Eastern Africa. Insect Mol. Biol..

[41] Donnelly MJ, Licht MC, Lehmann T (2001). Evidence for recent population expansion in the evolutionary history of the malaria vectors *Anopheles arabiensis* and *Anopheles gambiae*. Mol. Biol. Evol..

[42] Minakawa N (2005). Spatial distribution of anopheline larval habitats in Western Kenyan highlands: effects of land cover types and topography. Am. J. Trop Med. Hyg..

[43] Muturi EJ (2014). Population genetic structure of *Anopheles arabiensis* (Diptera: Culicidae) in a rice growing area of central Kenya. J. Med. Entomol..

[44] Gray EM, Bradley TJ (2005). Physiology of desiccation resistance in *Anopheles gambiae* and *Anopheles arabiensis*. Am. J. Trop Med. Hyg..

[45] Yamana TK, Eltahir EA (2013). Incorporating the effects of humidity in a mechanistic model of *Anopheles gambiae* mosquito population dynamics in the Sahel region of Africa. Parasit. Vectors.

[46] Nkumama IN, O’Meara WP, Osier FH (2017). Changes in malaria epidemiology in Africa and new challenges for elimination. Trends Parasitol..

[47] Chen H (2014). Monooxygenase levels and knockdown resistance (*kdr*) allele frequencies in *Anopheles gambiae* and *Anopheles arabiensis* in Kenya. J. Med. Entomol..

[48] Severson DW, Crampton JM, Beard CB, Louis C (1997). RFLP analysis of insect genomes. The Molecular Biology of Insect Disease Vectors.

[49] Scott JA, Brogdon WG, Collins FH (1993). Identification of single specimens of the *Anopheles gambiae* complex by the polymerase chain reaction. Am. J. Trop. Med. Hyg..

[50] Zheng L, Benedict MQ, Cornel AJ, Collins FH, Kafatos FC (1996). An integrated genetic map of the African human malaria vector mosquito, *Anopheles gambiae*. Genetics.

[51] Oetting WS (1995). Linkage analysis with multiplexed short tandem repeat polymorphisms using infrared fluorescence and M13 tailed primers. Genomics.

[52] Excoffier L, Lischer HE (2010). Arlequin suite ver 3.5: a new series of programs to perform population genetics analyses under Linux and Windows. Mol. Ecol. Resour..

[53] Raymond M, Rousset F (1995). GENEPOP (version 1.2): population genetics software for exact tests and ecumenicism. J. Hered..

[54] Rousset F (2008). Genepop'007: a complete reimplementation of the Genepop software for Windows and Linux. Mol. Ecol. Resour..

[55] Keenan K, McGinnity P, Cross TF, Crozier WW, Prodöhl PA (2013). diveRsity: An R package for the estimation and exploration of population genetics parameters and their associated errors. Methods Ecol. Evol..

[56] Pritchard JK, Stephens M, Donnelly P (2000). Inference of population structure using multilocus genotype data. Genetics.

[57] Kopelman NM, Mayzel J, Jakobsson M, Rosenberg NA, Mayrose I (2015). Clumpak: a program for identifying clustering modes and packaging population structure inferences across K. Mol. Ecol. Resour.

[58] Bates D (2015). Package ‘lme4’. Convergence.

[59] Beerli P, Felsenstein J (2001). Maximum likelihood estimation of a migration matrix and effective population sizes in n subpopulations by using a coalescent approach. Proc. Natl. Acad. Sci..

[60] Cushman S, Storfer A, Waits L (2015). Landscape Genetics: Concepts, Methods, Applications.

[61] Roy J (2005). Hijmans, R. J., Cameron, S. E., Parra, J. L., Jones, P. G. & Jarvis, A. Very high resolution interpolated climate surfaces for global land areas. Int. J. Climatol. J. R. Meteor. Soc..

[62] Channan S, Collins K, Emanuel WR (2014). Global Mosaics of the Standard MODIS Land Cover Type Data.

[63] Friedl MA (2010). MODIS Collection 5 global land cover: algorithm refinements and characterization of new datasets. Remote Sens. Environ..

[64] Tatem AJ (2017). WorldPop, open data for spatial demography. Sci. Data.

[65] McRae BH, Dickson BG, Keitt TH, Shah VB (2008). Using circuit theory to model connectivity in ecology, evolution, and conservation. Ecology.

[66] Adamack AT, Gruber B (2014). PopGenReport: simplifying basic population genetic analyses in R. Methods Ecol. Evol..

[67] Peterman WE (2018). ResistanceGA: An R package for the optimization of resistance surfaces using genetic algorithms. Methods Ecol. Evol..

[68] Peterman WE (2019). A comparison of popular approaches to optimize landscape resistance surfaces. Landsc. Ecol..

[69] Oyler-McCance SJ, Fedy BC, Landguth EL (2013). Sample design effects in landscape genetics. Conserv. Genet..

