# Supplementary material for: Ecological drivers of genetic connectivity for African malaria vectors Anopheles gambiae and An. arabiensis
Source: Sci Rep. 2020 Nov 17;10:19946. doi: 10.1038/s41598-020-76248-2 (PMC7673128; doi:10.1038/s41598-020-76248-2)
Supplement: Supplementary file 1 — Supplementary Information. [file 41598_2020_76248_MOESM1_ESM.pdf]

Ecological Drivers of Genetic Connectivity for African Malaria Vectors *Anopheles gambiae* and *An. arabiensis*

Elizabeth Hemming-Schroeder, Daibin Zhong, Maxwell Machani, Hoan Nguyen, Sarah Thong, Samuel Kahindi, Charles Mbogo, Harrysone Atieli, Andrew Githeko, Tovi Lehmann, James W. Kazura, Guiyun Yan

Supplementary table 1. Locality information and sample size by species used for analyses. Sample size (n) indicates number of mosquitoes included in this study from a given study site.

						Sample size (n)	
Region	Site name	Site code	Longitude	Latitude	Elevation	*An. arabiensis*	*An. gambiae* s.s.
Western Kenya Lowlands	Port Bunyala	PB	34.012	-0.114	1139	52	55
	Kendu Bay	KB	34.629	-0.385	1150	44	0
	Homa Bay	HB	34.465	-0.543	1184	36	38
	Kanyawegi	KN	34.606	-0.122	1214	27	55
	Miwani	MW	34.943	-0.129	1161	40	0
Western Kenya Highlands	Emutete	EE	34.618	0.023	1520	0	45
	Mayanja	MA	34.578	0.513	1408	0	40
	Kaimosi	KI	34.899	0.156	1647	0	21
	Kamkuywa	KM	34.807	0.571	1487	24	0
Rift Valley	Marigat	MT	36.018	0.484	1004	36	0
	Kabarnet	KT	35.663	0.498	1150	44	0
Coastal Kenya	Junju	JU	39.742	-3.852	66	27	0
	Kakayuni	KK	40.039	-3.170	14	27	0
Total sample size (n)						357	254

Supplementary Table 2. Microsatellite markers used for *An. gambiae s.l.* genotyping (Zheng et al 1996[52]). Sample size (n) indicates the number of mosquitoes which were successfully amplified at a given locus.

Locus	Chromosome	Annealing (°C)	Sample size (n)	
			An. arabiensis	*An. gambiae*
AG2H143	2L	60	300	214
AG2H46	2R	55	252	197
45C1	3L	60	278	0
AG3H577	3L	60	281	215
29C1	3R	50	295	213
33C1	3R	55	0	214
AG3H249	3R	60	279	0

Supplementary Table 3. Genetic diversity indices for *Anopheles gambiae* and *An. arabiensis* in Kenya. A_R is allelic richness; H_O is observed heterozygosity; H_E is expected heterozygosity; F_{IS} is the fixation index.

		An. Arabiensis				***An. gambiae s.s.***			
**Region**	**Site**	A_R	H_O	H_E	F_{IS}	A_R	H_O	H_E	F_{IS}
Western lowland	PB	5.12	0.343	0.679	0.493	3.57	0.351	0.503	0.302
	HB	5.22	0.290	0.562	0.485	4.17	0.347	0.559	0.372
	KB	4.76	0.411	0.576	0.273	–	–	–	–
	KN	6.24	0.463	0.720	0.349	4.90	0.462	0.584	0.203
	MW	5.34	0.309	0.578	0.476	–	–	–	–
Western highland	KI	–	–	–	–	3.74	0.369	0.588	0.397
	KM	5.59	0.417	0.694	0.419	–	–	–	–
	EE	–	–	–	–	5.59	0.459	0.630	0.267
	MA	–	–	–	–	5.87	0.402	0.653	0.384
Rift Valley	KT	5.11	0.382	0.588	0.373	–	–	–	–
	MT	5.17	0.314	0.554	0.351	–	–	–	–
Coastal	KK	3.46	0.211	0.461	0.499	–	–	–	–
	JU	5.17	0.393	0.723	0.442	–	–	–	–

Supplementary Table 4. Median migration rates (M) among all pairwise populations in Kenya.

Anopheles gambiae			***Anopheles arabiensis***					
**Source**	**Target**	**Median M**	**Source**	**Target**	**Median M**	**Source**	**Target**	**Median M**
KN	MA	32.33	PB	KN	24.33	KT	MT	13
HB	MA	29	MT	KN	23.67	HB	JU	13
EE	MA	26.33	PB	JU	23.67	KB	PB	12.33
EE	KN	23.67	KT	HB	20.33	MT	HB	12.33
PB	MA	23	KB	JU	20.33	KB	KK	14.33
HB	EE	21.67	MW	KN	19.67	JU	KM	12.33
HB	KN	21	HB	KM	19	KN	MW	12.33
KN	EE	21	KB	HB	18.33	KK	MT	12.33
PB	HB	20.33	MT	KM	18.33	JU	KN	12.33
PB	EE	19.67	HB	KN	18.33	MT	KK	13.67
KN	PB	19.67	KM	KN	18.33	KK	JU	12.33
HB	PB	19	PB	HB	17	KT	KM	11.67
PB	KN	16.33	PB	KT	17	MW	KT	11.67
KN	MA	16.33	PB	KB	16.33	MW	KB	11.67
MA	KN	16.33	KT	JU	17	HB	KB	11.67
KN	HB	15.67	KT	MW	16.33	HB	MW	11.67
EE	HB	15.67	PB	MW	15.67	KN	MT	11.67
KN	KN	15.67	KB	KM	15	HB	MT	11.67
PB	KN	15	KB	KT	15	JU	MT	11.67
EE	KN	14.33	MT	JU	15.67	KK	KN	11.67
KN	KN	14.33	MT	MW	15	HB	KK	13
MA	PB	13.67	KB	MT	15	KM	JU	11.67
KN	EE	13	PB	MT	15	KT	PB	11
MA	KN	12.33	KT	KN	15	KM	HB	11
KN	HB	11.67	PB	KM	14.33	KN	KM	11
MA	EE	11	KN	KT	14.33	KM	KB	11
KN	PB	11	JU	MW	14.33	PB	KK	12.33
EE	PB	11	KT	KK	16.33	KN	JU	11
MA	HB	10.33	MW	HB	13.67	KM	PB	10.33
HB	KN	10.33	MW	KM	13.67	KK	PB	10.33
			KK	KM	13.67	KN	HB	10.33
			HB	KT	13.67	JU	HB	10.33
			KK	MW	13.67	KK	HB	10.33
			MW	MT	13.67	MT	KT	10.33
			KM	MT	13.67	JU	KT	10.33
			MW	KK	15.67	KK	KB	10.33
			KB	KN	13.67	KM	MW	10.33
			KN	PB	13	KM	KK	11.67
			MW	PB	13	MT	PB	9.67
			HB	PB	13	JU	PB	9.67
			MW	JU	13.67	KM	KT	9.67
			KK	KT	13	MT	KB	9.67
			KT	KB	13	KN	KB	9.67
			JU	KB	13	KN	KK	11
			KB	MW	13	JU	KK	9.67

Supplementary Table 5. Model selection results of for linear mixed-effects models optimized on pairwise genetic differentiation (D_{PS}) in ResistanceGA.
Abbreviations: K, number of parameters in the mixed effects model; Avg., averaged over 1000 bootstrap iterations; ΔAICc, Difference in Akaike information criterion from the lowest AIC model; rank, model ranking; top model, percentage of the bootstrap iterations that a model was the top model.

Model	K	Avg. ΔAIC	Avg. rank	Top model (%)
Anopheles arabiensis				
1) Cropland	2	0	1.964	35.1
2) Tree Cover	2	0.197	1.545	61.2
3) Temperature	2	1.550	3.084	0.0
4) Geographic Distance	1	2.017	3.407	3.7
Anopheles gambiae				
1) Temperature	2	0	1.897	31.4
2) Tree Cover	2	0.129	2.047	44.5
3) Precipitation	2	1.803	2.736	16.2
4) Geographic Distance	1	4.667	3.320	7.9

Supplementary Table 6. Pearson correlation coefficients for input raster files used in landscape genetic analyses. Upper triangle represents correlation coefficients for rasters used for *Anopheles arabiensis* analysis*.* Lower triangle represents correlation coefficients for rasters used for *Anopheles gambiae*

	Average Temperature	Annual Precipitation	Human Population Density	Cropland	Road Proximity	Percent Tree Cover
Average Temperature	--	0.139	0.150	-0.009	-0.053	-0.386
Annual Precipitation	-0.407	--	0.359	-0.189	-0.342	0.285
Human Population Density	-0.095	0.283	--	-0.124	-0.250	-0.086
Cropland	0.090	-0.241	-0.063	--	0.222	0.134
Road Proximity	0.159	-0.497	-0.237	0.340	--	-0.058
Percent Tree Cover	-0.531	0.245	-0.085	0.172	0.103	--

Supplementary Table 7. Pairwise F_{ST} (lower) and D_{PS} (upper) values for *An. arabiensis* populations used for landscape genetic analysis.

	KT	KB	MT	PB	KN	MW	HB	KM
KT	—	0.4760	0.4997	0.3750	0.3550	0.5109	0.4157	0.4682
KB	0.1669	—	0.4504	0.3209	0.3386	0.4567	0.4459	0.3799
MT	0.2702	0.2312	—	0.3690	0.4338	0.2825	0.5417	0.4241
PB	0.1299	0.0774	0.2063	—	0.2314	0.4403	0.3136	0.2830
KN	0.0881	0.0476	0.1586	0.0158	—	0.4542	0.3758	0.3351
MW	0.2940	0.2473	0.0475	0.2642	0.1878	—	0.6253	0.4810
HB	0.1834	0.1602	0.2741	0.0892	0.0896	0.3067	—	0.3680
KM	0.1415	0.0575	0.1557	0.0308	0.0203	0.1989	0.0649	—

Supplementary Table 8. Pairwise F_{ST} (lower) and D_{PS} (upper) values for *An. gambiae* populations used for landscape genetic analysis.

	KI	PB	EE	MA	HB	KN
KI	—	0.5811	0.3455	0.3922	0.4249	0.4399
PB	0.2595	—	0.4735	0.4118	0.3963	0.5532
EE	0.0584	0.1654	—	0.2625	0.2997	0.2783
MA	0.1130	0.1254	0.0354	—	0.3798	0.3832
HB	0.1141	0.1305	0.0434	0.0691	—	0.4072
KN	0.1255	0.2295	0.0447	0.0826	0.1280	—

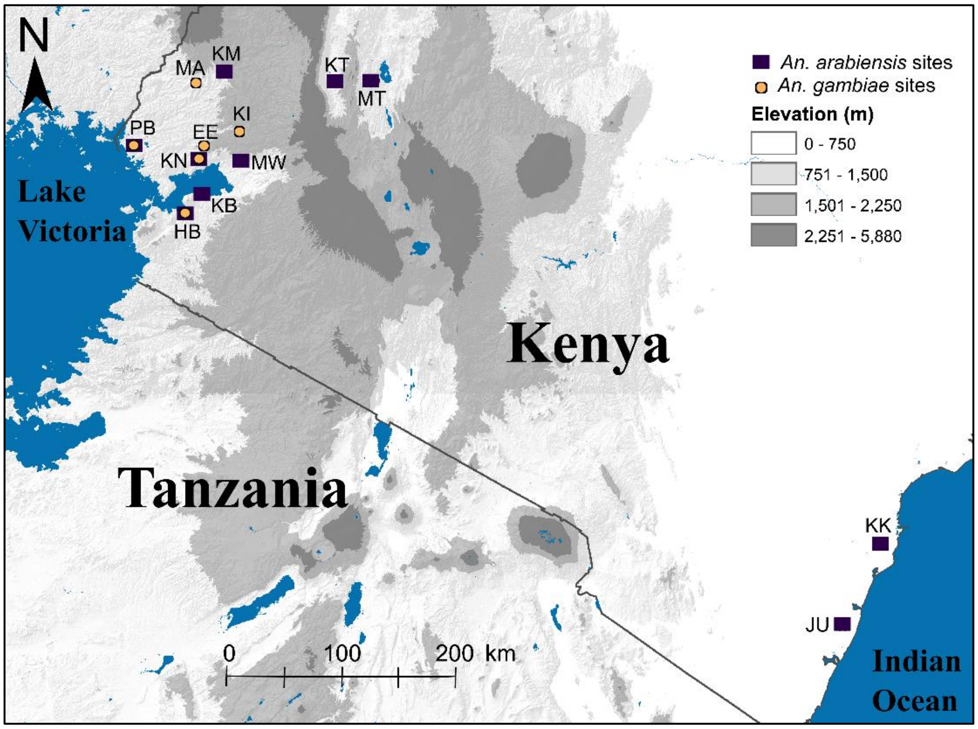

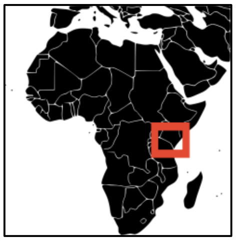

ID	Site Name
EE	Emutete
HB	Homa Bay
JU	Junju
KB	Kendu Bay
KI	Kaimosi
KK	Kakayuni
KM	Kamkuywa
KN	Kanyawegi
KT	Kabarnet
MA	Mayanja
MT	Marigat
MW	Miwani
PB	Port Bunyala

Supplementary Figure 1. Locations of study sites for *Anopheles gambiae* and *Anopheles arabiensis* across Kenya. The elevation map was created from Shuttle Radar Topography Mission (SRTM) data in ArcMap 10.6.1 (https://desktop.arcgis.com/en/arcmap/).

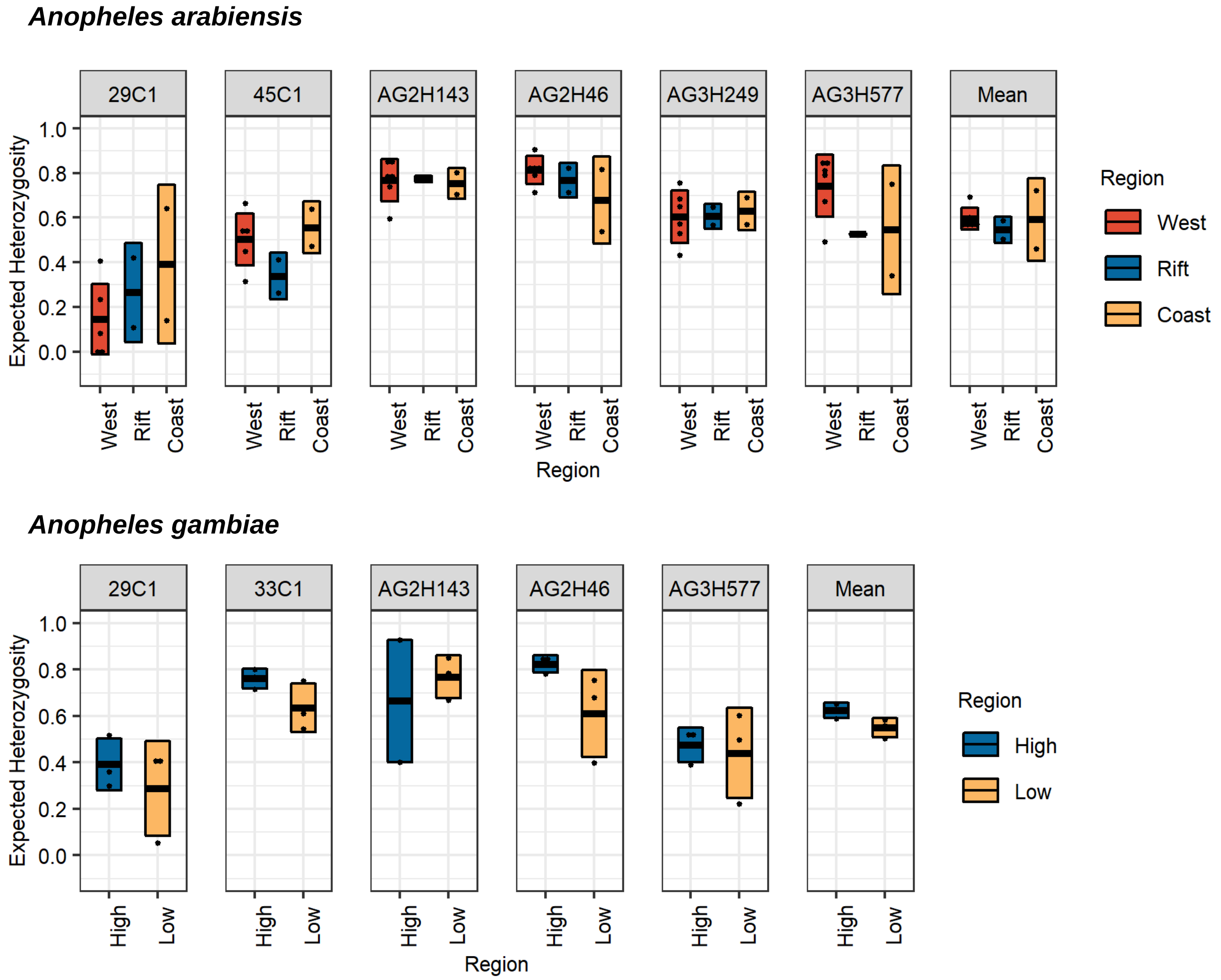

Supplementary Figure 2. Comparison of genetic diversity (expected heterozygosity) among regions for *Anopheles arabiensis* and *An. gambiae* by microsatellite locus. Dots represent individual populations. Bars indicate one standard deviation from the mean. No significant differences were observed between regions for either species (unpaired t-test; $P > 0.05$).

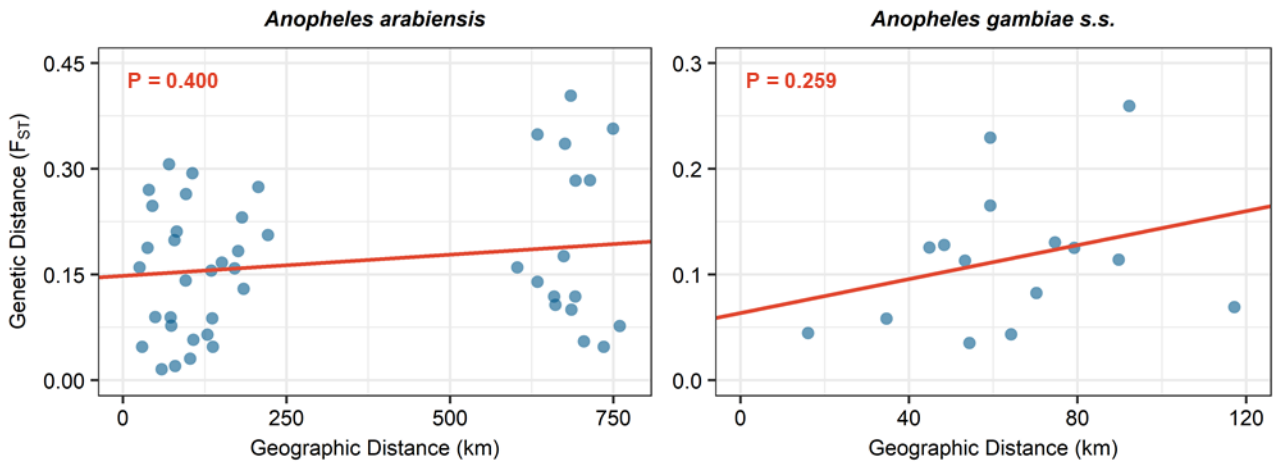

Supplementary Figure 3. Isolation-by-distance plots for *Anopheles arabiensis* and *Anopheles gambiae.*

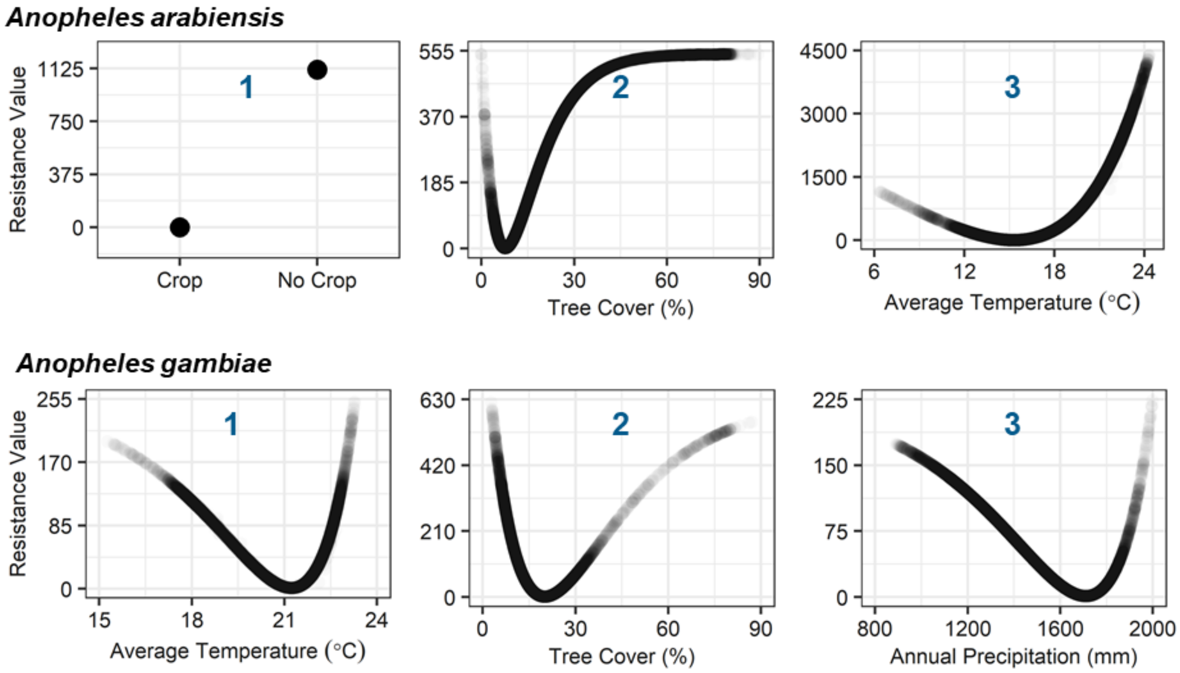

Supplementary Figure 4. Response curves signifying the relationship between ecological variables and landscape resistance to gene flow in the three highest performing single-surface models for *Anopheles gambiae* and *An. arabiensis* by D_{PS}. Blue number in plot indicates model ranking by Avg. ΔAIC value.

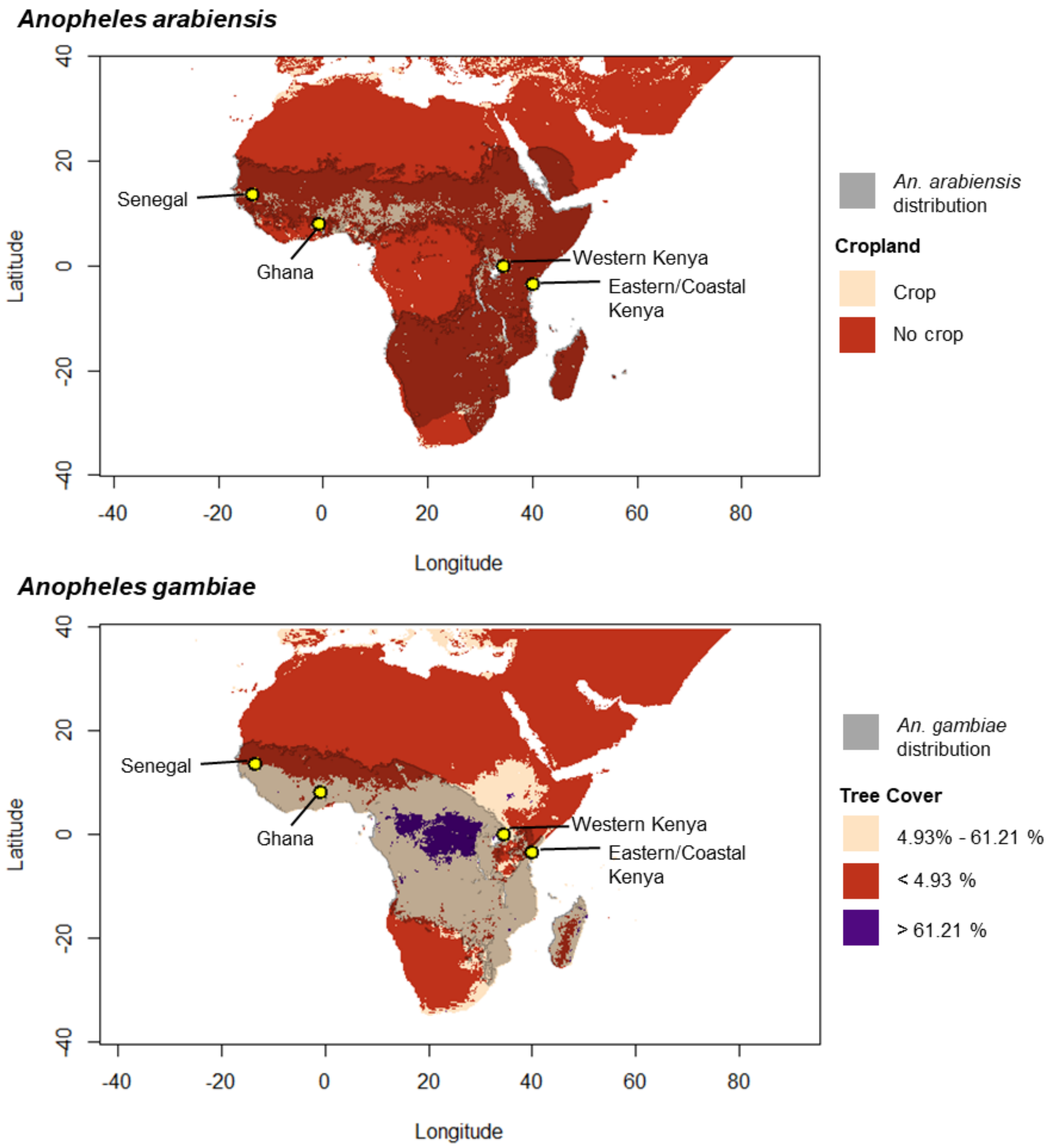

Supplementary Figure 5. Landscape resistance map for *Anopheles arabiensis* and *gambiae* across Africa based on the landscape in 2001. For *An. arabiensis,* red indicates high landscape resistance (areas with no crops) and light gold represents low landscape resistance (areas with crops or crop/natural vegetation mosaic). For *An. gambiae,* red and purple represent the areas of highest 30% landscape resistance (tree cover < 4.93% and > 61.21%, respectively). Light gold represents lowest 70% landscape resistance (tree cover between 4.93 and 61.21%). Vector distributions were obtained from The Malaria Atlas Project. The maps were created in R 3.6.0 (https://www.r-project.org/rdata).

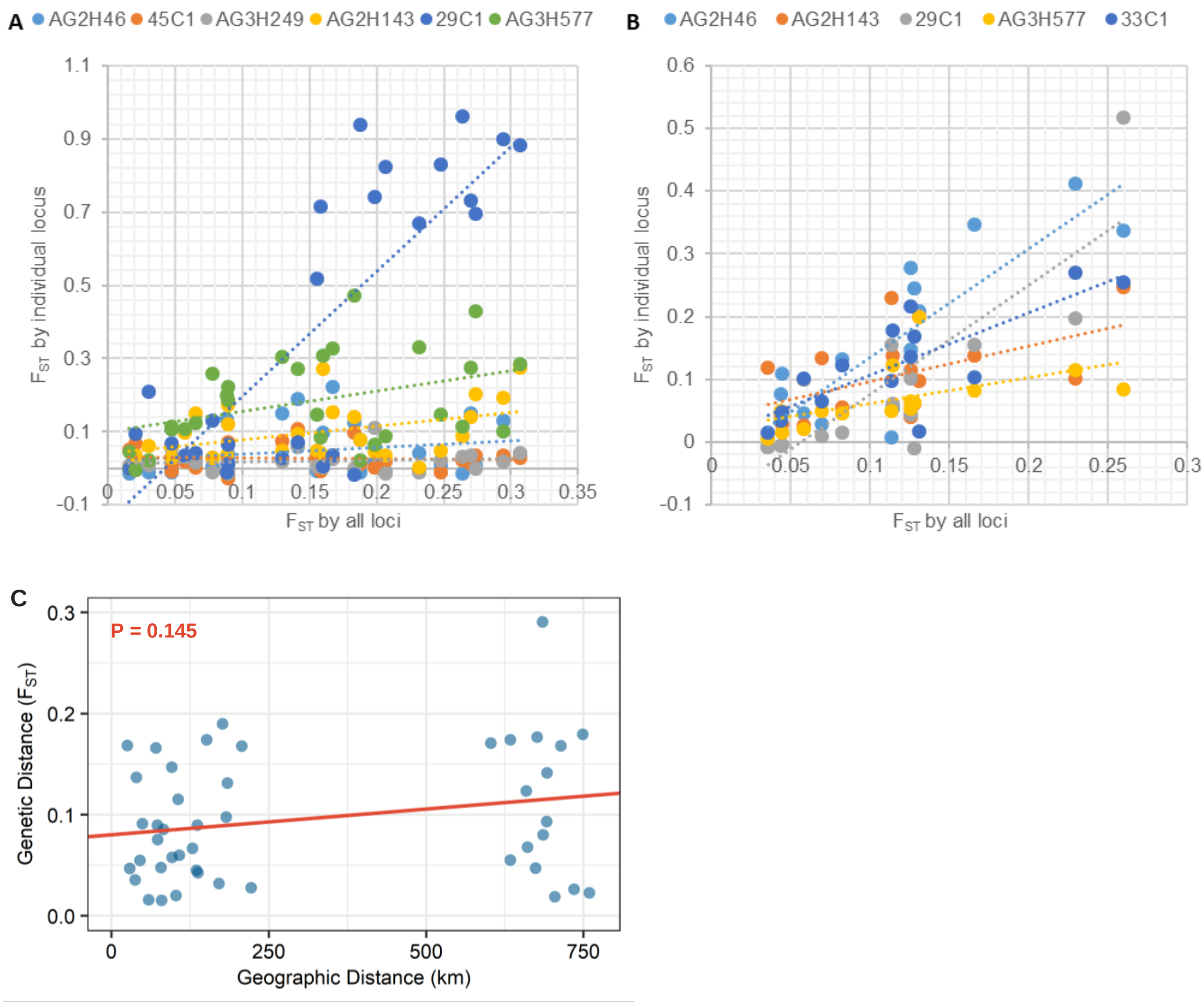

Supplementary Figure 6. Evaluation of locus-specific effects to overall F_{ST} values used in landscape genetic analyses. A) Correlation of F_{ST} by loci to overall F_{ST} for *Anopheles arabiensis.* Dots represent individual pairwise F_{ST} values. Dotted lines indicate linear trend lines and are colored to match the respective locus. B) Correlation of F_{ST} by loci to overall F_{ST} for *Anopheles gambiae.* Dots represent individual pairwise F_{ST} values. Dotted lines indicate linear trend lines and are colored to match the respective locus. C) Isolation-by-distance plot for *Anopheles arabiensis* based on F_{ST} calculated without the 29C1 locus.

Supplementary File 1. Raw microsatellite data for *Anopheles arabiensis*

POP_ID	AG2H46_1	AG2H46_2	45C1_1	45C1_2	AG3H249_1	AG3H249_2	AG2H143_1	AG2H143_2	29C1_1	29C1_2	AG3H577_1	AG3H577_2
KT	176	176	176	180	129	133	174	188	163	163	101	101
KT	176	176	174	174	129	135	174	182	163	163	101	101
KT	182	182	174	174	-9	-9	174	174	163	163	99	99
KT	182	182	170	174	139	139	141	141	-9	-9	99	101
KT	182	182	174	174	129	133	174	184	163	163	-9	-9
KT	176	186	174	174	-9	-9	180	180	163	163	-9	-9
KT	176	186	174	174	129	129	184	184	163	163	-9	-9
KT	180	180	170	174	129	133	184	176	163	163	101	101
KT	176	176	174	174	127	129	182	182	163	163	101	101
KT	184	184	174	174	129	129	182	182	163	163	-9	-9
KT	-9	-9	174	174	129	133	174	178	163	163	-9	-9
KT	-9	-9	174	174	129	129	174	178	-9	-9	101	101
KT	-9	-9	174	174	125	129	174	174	163	163	103	103
KT	-9	-9	172	172	129	133	174	174	163	163	103	103
KT	-9	-9	170	176	125	129	174	174	-9	-9	111	115
KT	180	180	174	174	-9	-9	174	174	163	163	-9	-9
KT	176	180	174	174	-9	-9	174	174	163	163	101	131
KT	-9	-9	174	174	-9	-9	184	184	163	163	101	101
KT	176	176	174	174	-9	-9	176	176	163	163	-9	-9
KT	176	176	-9	-9	-9	-9	176	184	163	163	101	103
KT	-9	-9	-9	-9	133	137	176	184	163	163	101	101
KT	176	176	174	174	127	127	176	184	163	163	101	101
KT	176	180	174	174	-9	-9	174	174	163	163	101	101
KT	176	176	174	174	-9	-9	174	180	160	160	-9	-9
KT	178	188	174	174	129	133	184	184	163	163	-9	-9
KT	178	178	174	174	127	129	184	184	-9	-9	-9	-9
KT	176	176	174	174	-9	-9	174	174	163	163	-9	-9
KT	176	176	174	174	129	129	174	184	163	163	-9	-9
KT	176	176	174	180	-9	-9	-9	-9	163	166	101	101
KT	176	184	174	174	129	129	184	184	163	166	99	99
KT	176	192	174	174	131	135	174	174	-9	-9	101	101
KT	176	176	174	174	125	129	174	184	163	163	-9	-9
KT	180	180	174	174	125	129	174	184	163	163	113	113
KT	-9	-9	174	174	129	133	174	174	163	163	101	101
KT	-9	-9	172	174	129	129	176	180	163	163	101	105
KT	-9	-9	174	174	129	129	176	186	163	163	99	101
KT	176	182	174	174	-9	-9	176	204	163	163	101	101
KT	-9	-9	174	174	-9	-9	174	184	163	163	101	101
KT	182	182	174	174	125	129	184	184	163	163	-9	-9

KT	-9	-9	174	178	129	135	-9	-9	163	163	103	103
KT	182	182	174	178	129	133	176	180	-9	-9	-9	-9
KT	-9	-9	174	174	129	129	174	180	-9	-9	101	101
KT	176	188	174	174	129	133	-9	-9	-9	-9	101	101
KT	180	180	174	174	-9	-9	174	182	163	163	101	101
KB	-9	-9	-9	-9	133	133	172	174	163	163	117	117
KB	178	178	174	174	129	129	172	174	163	166	-9	-9
KB	186	186	174	178	129	131	-9	-9	163	163	117	117
KB	186	186	174	178	-9	-9	172	182	163	163	117	117
KB	178	178	170	174	129	129	172	182	163	166	117	123
KB	178	186	170	170	129	129	172	182	163	166	-9	-9
KB	178	178	174	174	-9	-9	174	174	163	163	117	117
KB	178	184	174	174	129	129	-9	-9	163	166	117	117
KB	178	186	174	174	131	137	174	182	163	163	117	117
KB	-9	-9	174	174	129	131	172	182	163	163	113	121
KB	190	190	174	174	129	129	-9	-9	163	163	-9	-9
KB	-9	-9	174	174	129	131	174	178	163	166	121	125
KB	178	182	174	174	131	131	-9	-9	166	166	113	123
KB	-9	-9	174	174	129	129	174	174	163	166	123	123
KB	178	188	170	174	129	129	174	188	163	163	-9	-9
KB	178	182	-9	-9	129	141	172	182	163	163	117	117
KB	-9	-9	174	174	129	129	178	182	163	166	-9	-9
KB	186	186	-9	-9	129	135	-9	-9	163	163	133	133
KB	178	178	174	174	129	129	172	178	163	163	-9	-9
KB	178	178	174	174	129	133	172	182	163	163	115	115
KB	178	178	174	174	129	129	178	182	163	163	115	115
KB	178	190	170	170	129	133	-9	-9	163	163	-9	-9
KB	176	176	174	178	129	129	172	174	163	163	115	125
KB	186	186	174	174	129	133	172	182	163	163	-9	-9
KB	178	190	170	170	129	129	172	178	163	163	-9	-9
KB	178	190	174	176	127	127	174	182	163	163	119	119
KB	178	186	174	178	137	137	172	182	163	163	115	125
KB	176	176	172	174	131	131	174	182	163	163	-9	-9
KB	182	184	172	174	129	137	174	174	163	163	113	113
KB	186	186	174	174	129	131	172	172	163	166	125	125
KB	182	184	174	174	129	131	172	178	163	163	117	117
KB	178	178	170	174	131	131	172	186	-9	-9	-9	-9
KB	178	178	170	170	129	129	172	172	163	163	115	117
KB	178	178	172	174	-9	-9	172	174	-9	-9	115	117
KB	178	186	174	174	129	129	178	182	-9	-9	115	117
KB	178	178	174	178	129	129	172	172	163	163	-9	-9
KB	-9	-9	170	174	129	129	172	172	163	166	117	117
KB	182	184	-9	-9	129	129	172	178	163	163	-9	-9

KB	-9	-9	174	174	129	129	172	178	163	163	-9	-9
KB	178	178	174	174	-9	-9	172	174	163	163	113	113
KB	192	192	174	174	-9	-9	174	182	163	163	113	113
KB	-9	-9	174	174	129	133	-9	-9	163	163	115	115
KB	-9	-9	170	174	129	129	-9	-9	163	163	115	123
KB	-9	-9	174	174	129	129	172	182	163	163	-9	-9
MT	-9	-9	174	174	131	131	178	178	-9	-9	103	103
MT	184	184	-9	-9	129	129	178	178	160	160	103	103
MT	-9	-9	174	174	129	129	172	178	-9	-9	103	103
MT	-9	-9	174	174	129	129	-9	-9	160	160	101	103
MT	-9	-9	-9	-9	129	137	176	176	160	160	101	101
MT	-9	-9	174	174	129	129	172	178	142	160	-9	-9
MT	178	186	174	174	129	131	-9	-9	160	160	101	103
MT	186	186	-9	-9	129	129	172	176	160	160	103	103
MT	-9	-9	174	174	129	131	-9	-9	160	160	103	103
MT	186	186	174	174	-9	-9	-9	-9	160	166	103	117
MT	178	178	174	174	-9	-9	172	182	160	166	105	105
MT	-9	-9	174	174	-9	-9	172	176	160	160	101	103
MT	-9	-9	178	178	129	131	-9	-9	160	160	103	103
MT	-9	-9	172	176	129	131	172	176	160	160	101	103
MT	178	182	-9	-9	131	135	182	182	142	160	103	103
MT	178	182	170	176	-9	-9	172	182	163	163	103	103
MT	178	178	170	174	-9	-9	174	182	160	160	101	103
MT	-9	-9	174	174	131	135	172	174	160	160	101	103
MT	180	188	174	178	127	131	176	176	160	160	101	103
MT	-9	-9	178	178	129	129	172	180	142	142	-9	-9
MT	174	180	174	174	129	133	172	172	160	160	103	103
MT	-9	-9	174	174	129	129	172	182	-9	-9	103	103
MT	-9	-9	-9	-9	129	133	172	172	145	145	103	103
MT	182	182	170	174	131	131	-9	-9	142	142	-9	-9
MT	180	180	170	174	-9	-9	172	172	142	142	-9	-9
MT	180	180	174	174	129	131	-9	-9	160	160	105	117
MT	-9	-9	-9	-9	129	129	174	174	160	160	101	101
MT	-9	-9	174	174	129	131	-9	-9	160	160	101	101
MT	178	182	174	174	129	133	-9	-9	160	160	-9	-9
MT	178	188	174	174	129	129	172	182	-9	-9	103	103
MT	178	188	174	178	129	135	172	174	160	160	103	103
MT	178	182	170	174	-9	-9	174	174	-9	-9	103	103
MT	-9	-9	174	174	129	133	174	174	-9	-9	103	103
MT	-9	-9	174	174	129	129	178	178	-9	-9	101	101
MT	180	180	174	174	129	129	172	178	-9	-9	-9	-9
MT	-9	-9	-9	-9	129	129	172	182	160	160	101	101
PB	178	184	-9	-9	129	131	170	182	163	163	103	103

PB	180	186	174	174	129	129	172	182	163	163	103	117
PB	178	178	174	174	129	129	172	182	163	163	103	117
PB	178	184	174	174	-9	-9	174	182	163	163	103	103
PB	178	178	170	174	131	131	172	176	163	163	-9	-9
PB	178	178	174	174	-9	-9	172	182	163	163	103	103
PB	-9	-9	174	174	129	133	182	182	163	163	103	103
PB	-9	-9	170	174	129	129	174	174	163	163	103	103
PB	190	190	174	174	-9	-9	172	182	163	163	-9	-9
PB	186	186	-9	-9	129	129	172	172	163	163	103	105
PB	-9	-9	174	174	133	133	174	174	-9	-9	103	103
PB	182	186	174	174	129	129	168	172	163	163	101	105
PB	182	182	170	170	129	131	172	172	163	163	101	105
PB	178	186	-9	-9	-9	-9	176	176	163	163	101	101
PB	178	178	174	174	129	129	174	182	163	163	101	119
PB	180	180	-9	-9	137	137	174	174	163	163	101	103
PB	190	190	-9	-9	129	129	172	172	163	163	103	119
PB	178	186	178	178	129	131	174	174	163	163	103	103
PB	180	186	-9	-9	-9	-9	174	174	163	163	101	103
PB	180	186	-9	-9	135	141	174	178	163	163	103	103
PB	186	186	-9	-9	129	129	178	178	163	163	103	119
PB	176	176	-9	-9	129	129	172	172	-9	-9	103	119
PB	182	182	-9	-9	129	129	-9	-9	163	163	103	103
PB	178	190	176	176	129	131	-9	-9	-9	-9	103	103
PB	-9	-9	176	176	-9	-9	182	182	163	163	105	105
PB	-9	-9	176	176	131	133	180	180	163	163	105	105
PB	180	180	-9	-9	129	129	174	180	163	163	-9	-9
PB	182	182	-9	-9	133	135	174	178	163	163	105	105
PB	182	182	176	176	129	129	174	178	163	163	105	105
PB	178	178	176	176	129	129	174	184	163	163	-9	-9
PB	-9	-9	174	174	131	135	-9	-9	163	163	115	115
PB	178	178	174	174	129	129	-9	-9	163	163	103	107
PB	180	184	174	174	129	131	-9	-9	-9	-9	105	105
PB	178	178	-9	-9	133	133	184	184	163	163	105	105
PB	190	190	170	174	129	129	174	174	163	163	105	105
PB	184	184	174	174	-9	-9	176	176	-9	-9	105	105
PB	178	178	174	174	129	129	174	180	163	163	105	105
PB	186	186	-9	-9	129	129	174	182	-9	-9	105	105
PB	146	186	-9	-9	129	129	182	182	163	163	105	105
PB	176	186	176	178	129	129	174	182	163	163	103	107
PB	178	178	170	176	129	129	174	180	163	163	103	111
PB	178	178	174	174	129	131	174	174	163	163	103	111
PB	178	178	174	174	129	129	174	174	163	163	105	105
PB	176	180	-9	-9	131	131	170	170	163	163	105	105

PB	188	188	174	174	133	133	174	174	163	163	105	105
PB	180	180	-9	-9	-9	-9	174	174	163	163	103	103
PB	160	178	174	174	131	133	174	182	163	163	103	103
PB	160	178	174	174	129	131	174	184	163	163	103	103
PB	158	178	174	176	129	129	174	184	163	163	103	103
PB	184	184	-9	-9	129	133	172	182	-9	-9	103	103
PB	-9	-9	174	180	-9	-9	172	172	163	163	101	103
PB	178	184	-9	-9	131	131	172	172	163	163	103	103
KN	182	182	174	174	131	141	170	182	163	163	103	103
KN	176	184	174	178	-9	-9	174	174	-9	-9	103	117
KN	178	186	168	174	131	131	174	174	-9	-9	-9	-9
KN	176	176	174	180	131	131	172	172	163	163	103	123
KN	174	178	174	174	129	129	172	172	-9	-9	103	121
KN	186	186	174	174	127	131	-9	-9	163	163	105	117
KN	178	178	174	174	129	129	-9	-9	163	163	117	117
KN	-9	-9	174	174	127	129	174	180	163	163	-9	-9
KN	180	184	174	174	131	131	166	176	163	163	103	103
KN	180	180	170	174	133	133	166	166	163	163	103	103
KN	178	190	174	174	135	147	-9	-9	163	163	101	117
KN	178	186	174	174	133	135	-9	-9	-9	-9	103	121
KN	-9	-9	174	174	125	135	172	182	163	163	101	101
KN	186	186	174	174	129	133	164	176	163	163	101	121
KN	178	178	170	174	-9	-9	-9	-9	163	163	105	123
KN	178	180	170	174	129	133	164	176	163	163	-9	-9
KN	174	174	174	174	129	133	-9	-9	-9	-9	103	113
KN	192	192	174	174	129	133	176	184	-9	-9	123	123
KN	178	178	164	170	129	129	-9	-9	-9	-9	105	117
KN	178	178	174	174	129	129	-9	-9	-9	-9	105	105
KN	184	184	-9	-9	129	129	162	174	-9	-9	117	123
KN	178	184	174	174	129	145	-9	-9	-9	-9	105	119
KN	178	178	174	174	135	135	-9	-9	163	163	101	101
KN	176	176	174	174	129	137	-9	-9	-9	-9	103	115
KN	-9	-9	174	178	129	129	174	174	-9	-9	103	103
KN	-9	-9	174	174	129	133	174	174	-9	-9	101	127
KN	-9	-9	174	174	129	129	170	170	-9	-9	103	115
MW	-9	-9	174	174	-9	-9	172	172	160	160	101	105
MW	-9	-9	174	174	129	129	172	178	160	160	103	103
MW	178	178	-9	-9	129	129	172	178	160	160	103	103
MW	178	182	174	178	123	123	178	182	160	160	103	103
MW	178	186	-9	-9	129	129	-9	-9	160	160	103	103
MW	190	190	174	180	-9	-9	-9	-9	160	160	121	121
MW	184	184	-9	-9	-9	-9	182	182	160	160	119	119
MW	180	182	-9	-9	125	129	172	172	160	160	103	103

MW	178	178	174	174	125	129	-9	-9	160	163	101	101
MW	-9	-9	170	178	129	129	172	172	160	160	-9	-9
MW	178	188	174	174	-9	-9	172	172	160	160	101	101
MW	-9	-9	170	170	123	125	182	182	-9	-9	101	101
MW	180	180	174	174	133	133	172	182	160	160	101	123
MW	176	176	170	170	-9	-9	172	172	160	160	101	103
MW	-9	-9	-9	-9	129	129	172	178	160	163	99	99
MW	178	182	174	174	-9	-9	-9	-9	160	160	101	103
MW	-9	-9	172	172	129	133	172	182	160	160	117	117
MW	-9	-9	172	172	-9	-9	170	170	160	160	99	99
MW	-9	-9	170	170	133	133	174	176	160	160	99	103
MW	178	178	-9	-9	133	133	170	180	-9	-9	123	123
MW	178	178	174	178	129	129	170	170	-9	-9	-9	-9
MW	182	186	174	174	129	129	170	180	160	163	101	117
MW	176	176	174	178	-9	-9	168	170	160	160	101	121
MW	-9	-9	174	174	129	129	170	172	160	160	-9	-9
MW	-9	-9	174	174	-9	-9	180	180	160	160	113	113
MW	-9	-9	174	180	129	133	-9	-9	160	160	103	119
MW	178	184	174	174	-9	-9	180	180	160	160	105	119
MW	192	192	-9	-9	-9	-9	178	180	160	160	99	101
MW	-9	-9	172	172	129	129	172	172	160	160	101	101
MW	178	182	174	174	129	129	-9	-9	160	160	99	99
MW	182	182	174	174	129	129	168	172	-9	-9	99	103
MW	180	186	174	174	129	129	180	180	160	160	101	101
MW	-9	-9	174	178	129	129	-9	-9	160	160	97	119
MW	178	186	174	174	129	129	-9	-9	160	160	97	97
MW	178	186	174	174	129	129	170	176	160	160	99	99
MW	182	186	174	174	129	129	-9	-9	160	160	115	115
MW	182	182	174	174	129	129	170	174	160	160	-9	-9
MW	-9	-9	174	174	129	129	172	172	160	160	97	97
MW	-9	-9	174	174	-9	-9	172	172	160	160	101	101
MW	178	186	-9	-9	-9	-9	172	172	160	160	99	103
HB	158	178	-9	-9	129	129	176	176	163	163	-9	-9
HB	158	180	170	170	143	143	176	176	163	166	105	105
HB	160	182	170	174	-9	-9	176	176	163	163	-9	-9
HB	-9	-9	170	172	133	145	174	180	163	166	-9	-9
HB	-9	-9	-9	-9	129	129	176	180	163	163	103	103
HB	164	188	174	174	129	129	174	180	163	163	103	103
HB	178	178	-9	-9	133	133	-9	-9	163	163	105	105
HB	-9	-9	174	174	131	131	176	180	163	163	105	117
HB	-9	-9	174	174	129	129	176	176	163	163	105	105
HB	180	180	174	174	135	135	175	180	163	163	105	105
HB	152	176	-9	-9	129	133	176	184	-9	-9	105	105

HB	-9	-9	-9	-9	129	135	174	174	163	163	105	117
HB	154	178	176	176	129	129	174	174	163	163	105	105
HB	180	180	-9	-9	129	129	174	174	-9	-9	105	105
HB	180	180	-9	-9	129	129	174	174	160	166	105	105
HB	158	182	-9	-9	129	129	174	174	-9	-9	105	105
HB	-9	-9	-9	-9	129	129	174	174	163	163	105	105
HB	178	192	-9	-9	-9	-9	174	174	163	163	105	105
HB	154	182	-9	-9	129	135	174	174	-9	-9	105	105
HB	-9	-9	176	176	129	137	176	176	163	163	-9	-9
HB	160	178	176	176	-9	-9	176	176	163	163	105	105
HB	-9	-9	170	174	-9	-9	176	184	163	163	107	107
HB	180	180	174	174	129	129	176	176	-9	-9	105	117
HB	164	190	-9	-9	-9	-9	176	176	163	163	105	117
HB	158	158	176	176	127	127	174	176	163	163	103	103
HB	190	190	176	176	131	131	176	176	-9	-9	105	117
HB	156	180	174	174	131	131	176	176	163	163	105	105
HB	188	188	174	174	-9	-9	176	176	163	163	105	105
HB	180	180	174	174	129	131	174	176	-9	-9	105	117
HB	186	186	-9	-9	129	129	176	176	-9	-9	105	117
HB	162	162	174	174	-9	-9	176	176	163	163	105	117
HB	152	178	174	174	129	129	176	182	-9	-9	105	117
HB	160	178	174	174	133	133	-9	-9	163	163	-9	-9
HB	-9	-9	174	174	129	129	176	176	-9	-9	105	117
HB	162	186	174	174	127	127	176	176	163	163	-9	-9
HB	-9	-9	170	174	127	127	172	176	163	163	-9	-9
KM	178	178	-9	-9	131	131	172	172	163	163	-9	-9
KM	178	178	-9	-9	129	133	176	176	163	163	101	103
KM	168	178	170	174	129	133	180	182	163	163	115	117
KM	178	186	174	174	129	129	-9	-9	166	166	-9	-9
KM	178	186	174	174	-9	-9	-9	-9	154	163	113	117
KM	178	190	174	174	129	129	-9	-9	163	163	-9	-9
KM	190	190	130	172	125	129	172	174	163	163	105	105
KM	180	186	130	130	-9	-9	172	174	163	163	-9	-9
KM	178	178	128	128	125	129	-9	-9	163	163	103	109
KM	186	186	-9	-9	125	125	176	176	163	163	103	103
KM	180	184	-9	-9	131	131	174	174	154	160	105	105
KM	-9	-9	170	174	131	131	172	172	160	160	-9	-9
KM	184	188	-9	-9	129	129	174	190	163	163	-9	-9
KM	-9	-9	170	174	131	131	176	180	160	160	103	103
KM	180	180	170	174	129	131	184	184	163	163	-9	-9
KM	178	184	-9	-9	129	129	180	180	163	163	-9	-9
KM	178	184	-9	-9	-9	-9	178	180	163	163	101	105
KM	-9	-9	132	144	-9	-9	178	180	163	166	105	105

KM	180	186	-9	-9	129	129	180	180	-9	-9	111	117
KM	180	180	174	174	129	129	186	186	163	163	-9	-9
KM	180	180	174	174	129	131	-9	-9	163	163	-9	-9
KM	-9	-9	174	176	131	131	180	180	163	163	103	117
KM	186	186	174	174	-9	-9	172	172	160	163	103	105
KM	178	178	-9	-9	129	133	176	180	163	163	117	117
JU	186	186	174	174	131	135	172	172	163	169	101	101
JU	180	180	174	174	129	129	174	182	169	169	101	103
JU	186	186	-9	-9	129	133	172	172	169	169	101	103
JU	176	180	168	178	129	133	174	182	-9	-9	-9	-9
JU	-9	-9	174	174	129	135	176	176	-9	-9	101	103
JU	-9	-9	-9	-9	129	133	176	182	166	166	101	101
JU	-9	-9	-9	-9	129	129	174	174	166	166	99	101
JU	-9	-9	174	174	129	129	174	174	163	166	103	103
JU	186	186	178	180	129	135	174	180	163	163	101	105
JU	188	188	170	180	-9	-9	174	174	163	163	-9	-9
JU	-9	-9	-9	-9	131	135	174	174	163	163	101	101
JU	-9	-9	170	174	129	133	174	180	-9	-9	99	101
JU	180	180	174	178	129	129	174	180	-9	-9	105	119
JU	182	184	174	174	133	133	-9	-9	163	166	105	105
JU	180	186	174	174	129	131	174	180	166	166	-9	-9
JU	176	176	174	180	133	133	-9	-9	163	163	101	105
JU	-9	-9	-9	-9	131	133	182	186	163	163	101	101
JU	178	178	176	176	131	133	178	178	-9	-9	101	105
JU	178	178	-9	-9	129	131	182	182	163	163	105	105
JU	178	178	-9	-9	-9	-9	182	182	163	166	105	105
JU	178	178	174	174	133	133	182	182	163	166	117	117
JU	-9	-9	-9	-9	129	137	172	172	166	166	115	115
JU	-9	-9	176	176	129	133	174	174	163	166	-9	-9
JU	180	180	174	174	129	133	-9	-9	166	166	-9	-9
JU	-9	-9	176	176	129	131	180	184	-9	-9	101	105
JU	176	176	168	174	129	129	172	172	-9	-9	-9	-9
JU	186	186	174	174	-9	-9	172	172	169	169	103	103
KK	180	180	-9	-9	-9	-9	172	172	160	160	101	101
KK	-9	-9	141	141	131	131	182	182	160	160	-9	-9
KK	-9	-9	141	141	-9	-9	172	182	160	160	101	103
KK	-9	-9	174	174	-9	-9	172	182	160	160	101	101
KK	-9	-9	141	155	-9	-9	172	182	160	160	101	105
KK	178	178	174	174	-9	-9	172	172	160	160	-9	-9
KK	-9	-9	141	174	129	129	-9	-9	160	160	101	101
KK	182	182	-9	-9	129	129	172	172	160	163	101	101
KK	-9	-9	174	174	-9	-9	170	170	160	160	101	113
KK	-9	-9	174	174	-9	-9	174	182	160	160	101	101

KK	-9	-9	174	174	131	133	174	182	160	160	101	101
KK	178	178	-9	-9	133	133	172	172	160	160	101	101
KK	-9	-9	174	174	133	133	182	182	160	160	-9	-9
KK	178	178	-9	-9	133	133	182	182	160	160	101	101
KK	180	180	-9	-9	131	133	174	174	160	160	-9	-9
KK	-9	-9	174	174	-9	-9	174	182	160	160	101	101
KK	178	178	-9	-9	129	129	-9	-9	160	160	101	101
KK	-9	-9	155	174	-9	-9	172	172	160	160	101	101
KK	178	178	174	174	-9	-9	174	174	160	163	115	115
KK	178	178	174	174	133	133	172	182	160	160	-9	-9
KK	-9	-9	151	174	-9	-9	172	172	160	163	101	101
KK	180	180	174	174	-9	-9	172	172	160	160	101	111
KK	-9	-9	174	174	133	133	170	172	160	160	101	101
KK	178	178	155	174	129	133	178	182	160	160	101	101
KK	178	178	151	174	133	133	180	180	160	160	-9	-9
KK	178	178	-9	-9	133	133	172	182	160	160	101	101
KK	182	182	-9	-9	-9	-9	172	172	160	163	115	115

Supplementary File 2. Raw microsatellite data for *Anopheles gambiae*

POP_ID	AG2H46_1	AG2H46_2	AG2H143_1	AG2H143_2	29C1_1	29C1_2	AG3H577_1	AG3H577_2	33C1_1	33C1_2
KI	176	176	-9	-9	-9	-9	113	115	177	177
KI	176	182	172	176	163	166	115	115	-9	-9
KI	176	182	176	176	166	166	115	115	-9	-9
KI	188	188	172	176	163	163	115	115	174	177
KI	178	184	176	176	163	166	115	115	177	177
KI	182	182	176	180	163	166	119	119	180	180
KI	186	186	176	176	166	166	115	115	180	180
KI	-9	-9	-9	-9	163	166	115	115	174	174
KI	176	176	176	180	-9	-9	115	115	-9	-9
KI	186	186	180	180	-9	-9	115	115	-9	-9
KI	182	182	176	176	166	166	113	115	177	177
KI	182	182	176	176	-9	-9	113	115	174	180
KI	182	182	176	182	-9	-9	119	119	180	180
KI	178	182	176	176	-9	-9	115	115	174	180
KI	-9	-9	176	176	163	163	119	119	165	180
KI	182	182	-9	-9	163	166	109	115	165	174
KI	182	186	176	180	163	166	119	119	-9	-9
KI	178	186	176	176	-9	-9	-9	-9	174	180
KI	178	182	176	176	-9	-9	-9	-9	180	180
KI	178	178	176	176	163	163	115	115	177	177
KI	184	184	-9	-9	166	166	109	115	-9	-9
PB	-9	-9	168	172	-9	-9	113	115	174	174
PB	-9	-9	170	170	-9	-9	113	115	162	162
PB	186	186	164	164	-9	-9	113	115	-9	-9
PB	186	186	168	168	-9	-9	113	115	-9	-9
PB	188	188	168	174	-9	-9	113	115	177	177
PB	-9	-9	168	174	-9	-9	113	115	162	177
PB	-9	-9	168	168	-9	-9	115	115	162	162
PB	184	186	164	164	-9	-9	115	115	162	162
PB	-9	-9	168	168	166	166	115	115	177	177
PB	186	186	-9	-9	-9	-9	115	115	162	162
PB	-9	-9	170	176	166	166	115	115	-9	-9
PB	-9	-9	170	176	166	166	115	115	162	162
PB	-9	-9	172	172	166	166	-9	-9	162	162
PB	186	186	172	172	166	166	-9	-9	162	162
PB	186	186	176	172	166	166	-9	-9	162	174
PB	-9	-9	170	174	166	166	-9	-9	162	162
PB	188	188	168	174	166	166	113	115	-9	-9
PB	188	188	168	174	166	166	113	115	-9	-9

PB	184	184	168	174	166	166	117	115	174	177
PB	186	186	170	176	166	166	115	115	162	162
PB	184	184	168	168	166	166	117	115	162	162
PB	-9	-9	168	168	-9	-9	117	115	162	174
PB	-9	-9	-9	-9	163	166	113	115	162	162
PB	186	186	162	168	166	166	113	115	162	174
PB	184	186	168	170	163	166	113	115	162	174
PB	184	186	166	176	166	166	113	115	162	162
PB	186	186	164	164	166	166	113	115	162	174
PB	184	184	-9	-9	166	166	113	115	174	174
PB	184	184	174	174	-9	-9	113	115	174	174
PB	186	186	170	176	166	166	115	115	162	177
PB	-9	-9	168	172	166	166	113	115	162	162
PB	184	186	176	176	-9	-9	113	115	162	174
PB	184	186	172	174	-9	-9	113	115	-9	-9
PB	-9	-9	176	176	166	166	113	115	162	174
PB	-9	-9	164	170	-9	-9	113	117	162	174
PB	186	186	-9	-9	166	166	113	117	162	174
PB	186	186	174	174	166	166	113	117	162	162
PB	186	186	170	170	166	166	113	117	162	168
PB	186	186	172	172	166	166	117	117	162	162
PB	186	186	170	176	166	166	115	115	162	162
PB	186	186	-9	-9	166	166	-9	-9	174	177
PB	186	186	166	172	166	166	-9	-9	177	177
PB	186	186	-9	-9	166	166	-9	-9	174	177
PB	186	186	-9	-9	166	166	-9	-9	177	177
PB	-9	-9	174	174	166	166	-9	-9	162	162
PB	186	186	172	176	166	166	-9	-9	162	174
PB	186	186	166	166	166	166	-9	-9	162	174
PB	186	186	166	170	166	166	113	117	174	174
PB	186	186	172	172	-9	-9	117	117	174	174
PB	186	186	170	176	166	166	115	115	174	177
PB	186	186	172	172	-9	-9	117	117	162	174
PB	186	186	166	172	-9	-9	117	117	162	162
PB	-9	-9	170	176	-9	-9	115	115	162	165
PB	186	186	164	170	166	166	115	115	-9	-9
PB	186	186	168	168	166	166	115	115	177	177
EE	-9	-9	172	176	166	166	115	115	174	177
EE	-9	-9	172	176	163	166	115	115	174	174
EE	184	184	172	172	166	166	115	115	177	177
EE	-9	-9	172	172	-9	-9	115	115	174	174
EE	178	190	-9	-9	-9	-9	115	115	162	174
EE	-9	-9	176	176	163	166	-9	-9	162	174

EE	178	178	176	182	166	166	115	115	174	183
EE	182	190	176	176	166	166	115	115	174	177
EE	180	188	154	176	166	166	115	115	171	174
EE	-9	-9	172	176	166	166	-9	-9	162	171
EE	180	184	176	176	166	166	115	115	171	174
EE	180	180	172	176	166	166	107	107	174	174
EE	180	186	-9	-9	163	163	115	115	168	174
EE	178	184	180	180	163	166	115	115	171	177
EE	182	182	180	180	163	166	115	115	174	177
EE	180	184	178	178	166	166	115	115	174	177
EE	180	180	176	176	166	166	115	117	162	174
EE	176	182	176	176	166	166	115	119	177	177
EE	176	182	176	176	166	166	-9	-9	177	177
EE	182	182	154	176	163	166	113	115	162	174
EE	-9	-9	156	176	166	166	105	117	174	177
EE	-9	-9	176	176	163	166	107	115	165	168
EE	180	184	176	176	166	166	103	109	174	174
EE	178	178	176	186	163	166	103	109	174	174
EE	182	188	168	176	163	166	109	109	174	174
EE	180	184	176	178	163	166	109	115	168	174
EE	178	178	176	180	163	166	109	115	-9	-9
EE	178	178	-9	-9	-9	-9	117	117	177	177
EE	182	182	176	176	163	166	115	115	168	168
EE	-9	-9	-9	-9	166	166	101	115	162	174
EE	-9	-9	-9	-9	166	166	115	115	162	177
EE	182	190	156	176	166	166	115	115	168	174
EE	-9	-9	178	178	-9	-9	115	115	162	162
EE	-9	-9	-9	-9	166	166	111	111	162	162
EE	-9	-9	-9	-9	163	166	115	115	180	180
EE	180	186	-9	-9	166	166	-9	-9	159	159
EE	-9	-9	176	176	163	166	111	115	-9	-9
EE	184	188	176	176	163	166	-9	-9	174	174
EE	180	184	174	174	166	166	115	115	165	174
EE	-9	-9	174	174	163	166	-9	-9	174	174
EE	184	188	178	178	163	166	-9	-9	-9	-9
EE	-9	-9	176	176	163	166	-9	-9	165	171
EE	-9	-9	176	176	166	166	-9	-9	174	177
EE	-9	-9	-9	-9	166	166	115	115	171	177
EE	-9	-9	-9	-9	166	166	115	115	162	162
MA	180	184	-9	-9	166	166	-9	-9	165	174
MA	182	182	-9	-9	166	166	115	115	171	171
MA	186	186	156	174	-9	-9	115	115	177	177
MA	182	182	166	190	163	163	113	115	171	177

MA	176	176	180	180	166	166	113	115	171	177
MA	178	180	-9	-9	163	166	115	115	168	174
MA	-9	-9	168	186	166	166	115	115	168	177
MA	-9	-9	184	184	163	166	115	115	162	171
MA	186	186	-9	-9	166	166	-9	-9	171	174
MA	-9	-9	172	182	166	166	115	115	162	162
MA	180	188	168	194	-9	-9	115	115	168	168
MA	184	184	168	168	166	166	115	115	174	174
MA	182	186	164	178	163	166	115	115	162	174
MA	178	178	164	178	163	166	115	115	174	174
MA	176	182	178	178	166	166	117	117	162	174
MA	182	182	168	178	166	166	117	117	174	174
MA	178	178	168	178	166	166	111	115	-9	-9
MA	182	184	176	176	166	166	-9	-9	165	177
MA	-9	-9	168	178	166	166	115	115	162	174
MA	182	184	-9	-9	163	163	115	115	-9	-9
MA	-9	-9	164	188	166	166	115	115	171	177
MA	-9	-9	164	174	166	166	-9	-9	162	171
MA	-9	-9	-9	-9	166	166	115	115	174	177
MA	176	180	164	180	163	163	115	115	-9	-9
MA	182	182	172	176	166	166	-9	-9	177	177
MA	178	178	180	180	166	166	115	117	-9	-9
MA	-9	-9	162	180	166	166	-9	-9	162	174
MA	-9	-9	166	178	166	166	-9	-9	168	174
MA	-9	-9	172	184	166	166	117	117	-9	-9
MA	180	180	-9	-9	166	166	-9	-9	177	177
MA	178	186	168	192	166	166	-9	-9	177	177
MA	176	176	176	176	-9	-9	115	115	177	177
MA	184	184	164	182	163	163	-9	-9	168	168
MA	184	184	182	182	163	166	-9	-9	171	174
MA	-9	-9	182	182	166	166	-9	-9	171	177
MA	186	186	176	176	-9	-9	-9	-9	171	171
MA	-9	-9	-9	-9	166	166	115	115	177	177
MA	-9	-9	164	186	166	166	-9	-9	165	177
MA	180	184	158	174	166	166	113	115	-9	-9
MA	180	184	184	184	166	166	113	115	-9	-9
HB	182	182	174	174	166	166	115	115	174	174
HB	182	182	174	174	166	166	115	115	174	174
HB	-9	-9	174	174	166	166	103	115	162	174
HB	174	184	174	174	166	166	115	115	174	174
HB	174	174	174	174	166	166	115	115	156	165
HB	174	182	174	174	166	166	115	115	162	162
HB	174	182	172	176	166	166	115	115	162	162

HB	174	182	172	176	166	166	115	115	162	162
HB	184	184	172	176	166	166	115	115	171	174
HB	184	184	174	176	163	166	115	115	165	177
HB	184	184	172	176	163	166	115	115	162	177
HB	184	184	172	176	163	166	115	115	162	162
HB	186	186	172	176	163	166	115	115	177	177
HB	186	186	176	176	166	166	115	115	171	171
HB	184	184	172	176	163	166	115	115	162	162
HB	184	184	176	176	166	166	115	115	162	162
HB	184	184	174	176	163	166	103	105	174	174
HB	184	184	172	176	163	166	115	115	174	174
HB	186	186	170	170	163	166	115	115	174	174
HB	184	184	176	176	163	166	115	115	165	174
HB	186	186	174	174	166	166	115	115	159	162
HB	184	190	176	176	163	166	115	115	156	162
HB	182	188	176	176	166	166	115	115	174	174
HB	186	186	176	176	166	166	115	115	162	174
HB	186	186	-9	-9	163	166	115	115	171	174
HB	186	186	176	176	163	166	115	115	-9	-9
HB	186	186	176	176	163	166	115	115	162	186
HB	186	186	174	174	166	166	105	115	174	177
HB	182	182	176	176	163	166	115	115	-9	-9
HB	-9	-9	174	174	163	166	-9	-9	169	171
HB	-9	-9	172	172	163	166	103	105	-9	-9
HB	-9	-9	172	172	163	166	105	115	165	171
HB	-9	-9	172	176	163	166	105	105	-9	-9
HB	182	182	176	176	163	166	115	115	168	174
HB	182	182	172	176	166	166	115	115	162	162
HB	182	182	172	176	-9	-9	115	115	162	162
HB	182	182	172	176	163	166	115	115	162	162
HB	180	186	168	174	166	166	115	115	162	162
KN	178	178	176	176	166	166	115	115	174	174
KN	178	178	-9	-9	163	166	115	115	168	174
KN	-9	-9	170	176	163	166	103	115	168	174
KN	178	188	176	176	163	166	115	115	168	174
KN	178	178	176	176	-9	-9	115	115	174	174
KN	178	182	174	174	163	166	115	115	-9	-9
KN	178	188	170	170	163	163	107	115	-9	-9
KN	178	188	176	178	166	166	115	125	174	174
KN	178	186	182	182	166	166	103	115	174	174
KN	182	182	178	178	166	166	107	113	-9	-9
KN	178	188	158	176	166	166	115	125	-9	-9
KN	178	190	174	176	-9	-9	115	115	-9	-9

KN	178	190	174	178	-9	-9	115	115	-9	-9
KN	178	178	174	174	166	166	115	115	-9	-9
KN	178	178	180	180	166	166	107	123	171	177
KN	188	188	174	182	166	166	115	115	177	177
KN	178	188	176	178	166	166	103	115	-9	-9
KN	178	188	178	182	166	166	103	107	-9	-9
KN	176	188	182	182	166	166	103	107	174	174
KN	178	178	178	178	166	166	115	115	174	174
KN	178	178	174	176	163	163	115	115	174	174
KN	178	178	178	178	166	166	115	115	174	174
KN	178	188	178	178	166	166	103	103	174	174
KN	178	192	178	182	163	166	115	115	174	177
KN	188	188	174	174	163	166	115	115	174	177
KN	178	182	176	182	163	166	103	115	168	174
KN	178	178	176	182	163	166	115	115	168	174
KN	178	182	-9	-9	163	166	103	103	174	174
KN	178	190	-9	-9	166	166	115	115	168	174
KN	178	186	170	176	163	166	107	115	-9	-9
KN	178	182	-9	-9	166	166	103	103	168	168
KN	-9	-9	176	178	166	166	-9	-9	168	177
KN	182	190	176	176	166	166	115	115	174	177
KN	182	188	174	176	163	166	115	115	174	177
KN	178	178	-9	-9	-9	-9	115	115	168	174
KN	178	182	-9	-9	-9	-9	115	115	171	171
KN	178	178	176	178	166	166	101	115	-9	-9
KN	180	190	-9	-9	166	166	115	115	177	177
KN	178	188	174	180	166	166	115	115	-9	-9
KN	178	190	-9	-9	163	163	103	103	-9	-9
KN	-9	-9	176	176	166	166	115	115	174	174
KN	180	188	176	190	166	166	115	115	174	174
KN	180	186	-9	-9	166	166	105	115	174	174
KN	178	178	184	184	-9	-9	-9	-9	171	177
KN	178	178	-9	-9	163	166	103	115	174	174
KN	178	178	180	182	163	166	107	115	177	177
KN	176	176	176	182	163	166	107	115	171	174
KN	178	186	176	176	163	166	107	115	177	177
KN	178	186	176	176	163	166	103	115	174	174
KN	180	180	170	176	163	166	115	115	174	174
KN	178	186	176	176	163	166	115	119	174	174
KN	178	178	176	178	166	166	115	115	174	174
KN	178	186	172	180	163	166	115	115	174	174
KN	182	188	176	176	163	166	107	115	174	174
KN	178	188	176	184	163	163	115	115	174	174
